# Hypoxia enhances anti-fibrotic properties of extracellular vesicles derived from hiPSCs via the miR302b-3p/TGFβ/SMAD2 axis

**DOI:** 10.1186/s12916-023-03117-w

**Published:** 2023-10-31

**Authors:** Milena Paw, Agnieszka A. Kusiak, Kinga Nit, Jacek J. Litewka, Marcin Piejko, Dawid Wnuk, Michał Sarna, Kinga Fic, Kinga B. Stopa, Ruba Hammad, Olga Barczyk-Woznicka, Toni Cathomen, Ewa Zuba-Surma, Zbigniew Madeja, Paweł E. Ferdek, Sylwia Bobis-Wozowicz

**Affiliations:** 1 Faculty of Biochemistry, Biophysics and Biotechnology, Department of Cell Biology, Jagiellonian University, Kraków, Poland; 2 Doctoral School of Exact and Natural Sciences, Jagiellonian University, Kraków, Poland; 3 3Rd Department of General Surgery, Jagiellonian University - Medical College, Kraków, Poland; 4 Faculty of Biochemistry, Biophysics and Biotechnology, Department of Biophysics, Jagiellonian University, Kraków, Poland; 5 Małopolska Centre of Biotechnology, Jagiellonian University, Kraków, Poland; 6 Freiburg iPS Core Facility, Institute for Transfusion Medicine and Gene Therapy, Medical Center, University of Freiburg, Freiburg, Germany; 7 Center for Chronic Immunodeficiency (CCI), University of Freiburg, Freiburg, Germany; 8 Institute of Zoology and Biomedical Research, Department of Cell Biology and Imaging, Jagiellonian University, Kraków, Poland

**Keywords:** Extracellular vesicles, Induced pluripotent stem cells, Hypoxia, Low oxygen, Heart fibrosis, Therapy

## Abstract

**Background:**

Cardiac fibrosis is one of the top killers among fibrotic diseases and continues to be a global unaddressed health problem. The lack of effective treatment combined with the considerable socioeconomic burden highlights the urgent need for innovative therapeutic options. Here, we evaluated the anti-fibrotic properties of extracellular vesicles (EVs) derived from human induced pluripotent stem cells (hiPSCs) that were cultured under various oxygen concentrations.

**Methods:**

EVs were isolated from three hiPSC lines cultured under normoxia (21% O_2_; EV-N) or reduced oxygen concentration (hypoxia): 3% O_2_ (EV-H3) or 5% O_2_ (EV-H5). The anti-fibrotic activity of EVs was tested in an in vitro model of cardiac fibrosis, followed by a detailed investigation of the underlying molecular mechanisms. Sequencing of EV miRNAs combined with bioinformatics analysis was conducted and a selected miRNA was validated using a miRNA mimic and inhibitor. Finally, EVs were tested in a mouse model of angiotensin II-induced cardiac fibrosis.

**Results:**

We provide evidence that an oxygen concentration of 5% enhances the anti-fibrotic effects of hiPS-EVs. These EVs were more effective in reducing pro-fibrotic markers in activated human cardiac fibroblasts, when compared to EV-N or EV-H3. We show that EV-H5 act through the canonical TGFβ/SMAD pathway, primarily via miR-302b-3p, which is the most abundant miRNA in EV-H5. Our results show that EV-H5 not only target transcripts of several profibrotic genes, including *SMAD2* and *TGFBR2*, but also reduce the stiffness of activated fibroblasts. In a mouse model of heart fibrosis, EV-H5 outperformed EV-N in suppressing the inflammatory response in the host and by attenuating collagen deposition and reducing pro-fibrotic markers in cardiac tissue.

**Conclusions:**

In this work, we provide evidence of superior anti-fibrotic properties of EV-H5 over EV-N or EV-H3. Our study uncovers that fine regulation of oxygen concentration in the cellular environment may enhance the anti-fibrotic effects of hiPS-EVs, which has great potential to be applied for heart regeneration.

**Graphical Abstract:**

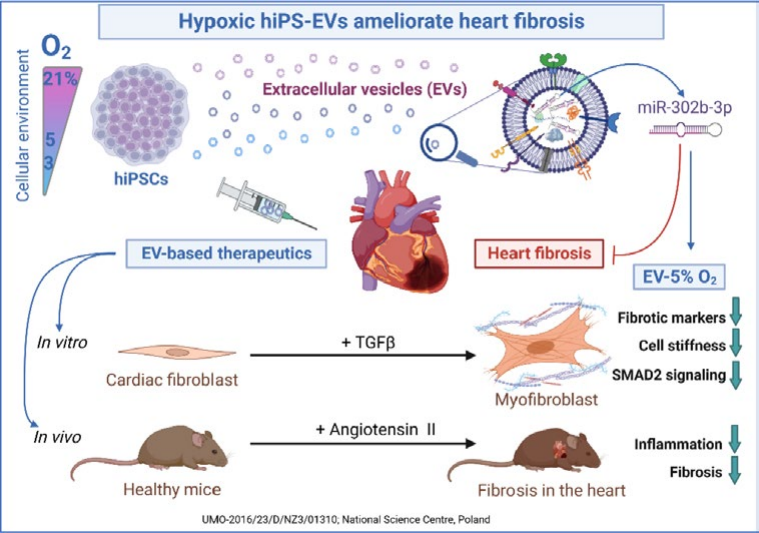

**Supplementary Information:**

The online version contains supplementary material available at 10.1186/s12916-023-03117-w.

## Background

The incidence of cardiovascular diseases has increased dramatically in the last decades and remains the predominant cause of mortality worldwide [1, 2]. Although myocardial dysfunction during heart diseases is often associated with impaired cardiomyocyte activity, cardiac fibrosis is the major cause of end-stage heart damage [3, 4]. Myocardial fibrosis is a complex process that results from abnormal healing of the heart tissue and ultimately leads to the formation of a non-functional scar, which hampers the activity of the entire organ. This involves both extracellular matrix overproduction and the activation of structural non-excitable fibroblasts to differentiate into contractile myofibroblasts, in a process called fibroblast-to-myofibroblast transition (FMT). Myofibroblasts are characterized by an increased expression of α-smooth muscle actin (α-SMA, encoded by the *ACTA2* gene) and the secretion of a number of pro-fibrotic proteins, such as collagens, fibronectin, or tenascin [5–7]. Transforming growth factor β (TGFβ) is the best-known fibrogenic cytokine described in fibrotic diseases, including heart fibrosis [6, 8, 9]. By enhanced activation of multiple signaling pathways, particularly those involving SMAD2/3 proteins, TGFβ is able to effectively induce the FMT machinery and propagate the profibrotic signaling cascade [3, 6].

Currently used therapeutic strategies targeting cardiac fibrosis such as β-blockers or cell-based therapies neither prevent the progression of cardiac fibrosis nor promote the functional recovery of the heart [10]. Therefore, innovative treatment strategies are an important unmet clinical need.

One of the novel classes of therapeutics which has gained considerable interest in recent years constitutes extracellular vesicles (EVs). EVs are nanometric circular structures secreted by virtually all types of cells under physiological and pathological conditions. They contain bioactive components derived from the parental cell, enclosed in a lipid bilayer that protects them against rapid degradation [11]. Based on their size and origin, EVs can be classified as exosomes, microvesicles (shedding vesicles), and apoptotic bodies. EVs are considered to be important mediators of intercellular communication and, due to their ability to transport bioactive molecules such as proteins, lipids, and various RNA molecules, they can influence the phenotype and properties of other cells [12]. In particular, mesenchymal stem/stromal cells (MSCs), cardiac progenitor cells (CPCs), cardiospheres, endothelial cells and pluripotent stem cells were shown to produce EVs with reparative capabilities, including anti-fibrotic activity [10, 13]. Owing to their unmatched functionality, biocompatibility, and efficiency in delivering components to target cells, EVs are regarded as new generation therapeutics in the treatment of a variety of human diseases [14].

We have previously shown that EVs derived from induced pluripotent stem cells (iPSCs) carry pro-regenerative potential, with respect to heart regeneration [15, 16]. Not only were they effective in transferring their cargo to primary cardiac stromal cells influencing their fate, but they also improved heart recovery post-myocardial infarction. Recent reports indicate the usefulness of hiPS-EVs in rejuvenation [17], immunomodulation [18], and cytoprotection [18–21]. Importantly, hiPS-EVs were also shown to exert anti-fibrotic function in the treatment of liver and pulmonary fibrosis [22, 23]. These data indicate that the spectrum of potential medical indications that can benefit from iPS-EV-based therapy is increasing. However, none of the available reports show the possibility of significant enhancement of the anti-fibrotic function of hiPS-EVs by modulating the cell culture environment of iPSCs, which we present in this study.

It is known that stem cells reside in hypoxic niches and that oxygen plays an important role in the regulation of cellular metabolism and function [24, 25]. In addition, reduced oxygen levels have been demonstrated to affect the cargo and activity of EVs derived from both normal and cancer cells [26, 27]. In particular, hypoxia was shown to increase the pro-angiogenic activity of EVs derived from various types of cells, including hiPSCs [28], embryonic stem cell-derived CPCs [29], MSCs [30, 31], and microglia [32], among others. However, the impact of low oxygen conditions on the anti-fibrotic function of hiPS-EVs and the underlying molecular mechanism has not been investigated so far.

Based on this knowledge, we selected oxygen conditions resembling physiological levels within the pluripotent stem cell niche, which were 5% O_2_ and 3% O_2_, along with 21% O_2_, as a standard cell culture setting. Conditioned media harvested from hiPSCs were processed to isolate therapeutic EVs, with the ultimate goal of ameliorating heart fibrosis. Importantly, we also elucidated the mechanisms mediated by hiPS-EVs, pointing to their miRNA cargo. Finally, we examined the anti-fibrotic function of hiPS-EVs in an in vivo model of angiotensin II-induced heart fibrosis.

## Materials and methods

### Cell culture

In this study, three iPSC lines were used: L1 — a previously published cell line [15]; L2 — episomal hiPSC line purchased from Gibco, #A18945; and L3 — cell line generated in the laboratory of Prof. Toni Cathomen – Medical Center – University of Freiburg, Freiburg, Germany. iPSCs were cultured in Essential8 medium (Gibco/Thermo Fisher Scientific), supplemented with antibiotics (Penicillin/Streptomycin; P/S; Gibco) on cell culture plates coated with human recombinant vitronectin (Gibco), in an atmosphere containing various values of oxygen concentration: 21% (normoxia; N) — in a standard cell incubator, or in the presence of a reduced oxygen concentration (hypoxia; H) — 5% O_2_ (H5) or 3% O_2_ (H3). Hypoxic conditions were obtained in an InvivO2 chamber (Ruskinn). Cells were passaged every 4 days using 0.5 mM EDTA solution (Gibco) and plated into new culture vessels coated with vitronectin in a ratio of 1: 6–8, with the addition of 10 µM ROCK kinase inhibitor (Y-27632; Millipore) for the first 24 h (h). Cultures were carried out in an atmosphere of 5% CO_2_, 70–90% humidity, with medium change every 24 h. The cells were adapted to each oxygen condition for 4 passages before starting the experiments.

Human dermal fibroblasts (hDF; ATCC, # PCS-201–012) were cultured in Dulbecco’s Modified Eagle’s Medium/High Glucose (DMEM/HG; Sigma-Aldrich/Merck), supplemented with 10% fetal bovine serum (FBS; Gibco) and 1% P/S in standard cell culture conditions (37 °C, 95% humidity, 5% CO_2_).

Human cardiac fibroblasts (hCF, Cell Applications/Merck, #306-05A) were cultured in Cardiac Fibroblast Growth Medium (Cell Applications, #316–500). Cells were grown under standard conditions. Fibrosis experiments were performed in Advanced DMEM/F12 medium (A/DMEM/F12; Gibco) containing 2% FBS, after adapting the cells to modified conditions.

### Isolation of EVs

Conditioned medium (CM) was harvested from iPSCs grown to a density of 70–90%, 24 h after the last medium change. EVs were isolated using the ultracentrifugation (UC) or ultrafiltration (UF) method combined with size-exclusion chromatography (SEC). Media were collected and frozen at -80°C until isolation. CM was sequentially centrifuged at 4°C at increasing speeds: 500 g, 8 min (to remove dead cells and cell debris) and 2000 g, 15 min (to remove apoptotic bodies). Then, EVs were obtained using either the UC or UF + SEC methods. Based on the UC method, EVs were collected at 100,000 g, 4°C for an hour, using the Sorvall WX 90 + Ultracentrifuge equipped with the T-865 fixed angle rotor (Thermo Fisher Scientific). The EVs pellet was washed with PBS and centrifuged again under the same conditions. The obtained EVs were suspended in PBS and frozen in aliquots at – 80°C. When EVs were obtained with the UF + SEC method, CM was concentrated in 15 ml filter tubes (Amicon/Merck) with a protein cut-off of 10, 50, and 100 kDa by centrifugation at 1800 g for 40–60 min at 4°C. Next, the supernatants were transferred to low-adhesive 1.5-ml tubes and kept on ice. Concentrated preparations were purified from proteins, lipids, and other small molecules using the EVs isolation column (qEV/70 nm, Izon), according to the manufacturer’s instructions. Briefly, the column was washed with PBS filtered through filters with a pore size of 0.22 µm. In the optimization stage, 0.5 ml fractions were collected from the column to 1.5 ml tubes by gravity flow, including the void volume (the first 3 ml), and were analyzed for the presence of EV particles and proteins. Subsequently, fractions 0.5–1.5 ml (after the void volume) were verified to contain EVs and were collected in a 1.5-ml low-adhesive tube in the rest of the experiments. EV preps were further concentrated using 4 ml concentrating tubes (Amicon) with a protein cut-off of 10 kDa. The resulting EV solution was aliquoted and stored at − 80 °C until use. Five independent isolations were performed to compare the results from the UF + SEC and UC methods. A sample of Essential8 medium was prepared the same way as EVs with the UF-10 kDa + SEC method, using 25 ml of medium.

To harvest EVs from hDF, cells were grown to 70–90% confluency. Next, cells were washed 2 × with PBS and a serum-free medium composed of DMEM/HG supplemented with 0.1% bovine serum albumin (BSA) was added for 48 h. CM was collected and processed with the UF-10 kDa + SEC method as described above.

### Measurement of EVs size and concentration

The size and concentration of EV particles were analyzed using the NanoSight apparatus (Malvern). The samples were diluted 1:1000 in PBS, or 1:100 for the analysis of EV fractions collected from the qEV/70 nm (Izon) column, to obtain the optimal particle density for measurement. Data were collected at camera level 11 and detection threshold 5. A 60-s video was recorded for each sample. Two EV batches were analyzed of each type of EVs (*n* = 6 for each oxygen condition, or *n* = 3 for EV fractions).

The amount of protein in the EV preparations was measured based on the reaction with bicinchoninic acid (BCA) (Invitrogen/Thermo Fisher Scientific), according to the manufacturer’s instructions.

### Transmission electron microscopy

Negative staining for EV was obtained by adsorbing 20 µl of EV suspended in PBS onto nickel-coated grids (Agar Scientific, Stansted, UK) for 30 min, followed by fixation for 5 min in 2.5% glutaraldehyde solution. After removing excess liquid with filter paper, the samples were stained with 2% uranyl acetate for 30 min, washed three times with distilled water for 1 min, and dried. Then, EVs were imaged with a JEOL JEM 2100HT (Jeol Ltd, Tokyo, Japan) transmission electron microscope (TEM) that was used at an accelerating voltage of 80 kV. Images were taken by using 4 k × 4 k camera (TVIPS) equipped with EMMENU software ver. 4.0.9.87.

### Cell metabolism assessment

Cell metabolic activity was measured with the Cell Counting Kit-8 (Sigma-Aldrich), upon stimulation with EVs. Briefly, hCFs were seeded on 96-well plates (2 × 10e3 cells per well) and were treated with EVs (2.5 × 10e4 EV particles/cell) for 24 h, after which the medium was replaced with a fresh medium. At indicated time points the CCK-8 reagent was added to cells for 2 h and the absorbance resulting from the activity of cellular dehydrogenases was measured in an Infinite M200 Microplate Reader (Tecan).

### In vitro model of fibrosis

Cells were seeded at a density of 5 × 10e4 cells/cm^2^ (for RNA and protein analyzes) or 8 × 10e3 cells/cm^2^ (for immunofluorescence) 24 h before starting the experiments in a complete cell culture medium. Then, the medium was replaced with A/DMEM/F12 containing 2% FBS, P/S, and TGFβ_1_ (TGFβ; Corning) at a concentration of 1 ng/ml for 6 h to induce fibrosis. Next, EVs from one cell line and one oxygen condition (hiPSC-L3, H5) were added to the cells at increasing concentrations: 1.25, 2.5, 5 × 10e4 particles/cell to optimize the dose. A concentration of 2.5 × 10e4/cell was used in the rest of the experiments. EVs from all three hiPSC lines and various oxygen concentrations (EV-N, EV-H5, EV-H3) were tested. hDF-EVs at the same dose (2.5 × 10e4/cell) were used for comparison. hCFs were exposed to EVs for 24 h after which the medium was removed. Fresh medium supplemented with TGFβ (1 ng/ml) was changed daily for the next 4 days, when the experiments were terminated.

### Immunofluorescence

The presence of pluripotency markers in hiPSCs and selected fibrotic proteins in hCFs were visualized by staining with specific antibodies. Cells were grown in glass bottom 24-well plates (Ibidi, # 82,406). After completion of the experimental procedure, cells were washed with PBS and then fixed with 3.7% formaldehyde solution (v/v) for 20 min at room temperature (RT). Next, cells were washed 3 × with PBS and permeabilized with 0.1% Triton X-100 solution in PBS (v/v) for 8 min. Again, cells were washed with PBS and incubated with 1% BSA solution in PBS (w/v) for 45 min at RT. Incubation with a primary antibody solution in 1% BSA was carried out overnight at 4°C. After washing thoroughly, cells were incubated with secondary antibodies for 45 min at RT. The list of antibodies is provided in Table 1. Cell nuclei were stained with DAPI (4′,6-diamidino-2-phenylindole, dihydrochloride) or Hoechst 33258 solution (1 µg/ml; Sigma-Aldrich), in case of hiPSCs or hCFs, respectively. hCFs were additionally stained with AF546 conjugated phalloidin (1:40; Invitrogen, #A22283), where indicated. Cells were washed with distilled water and analyzed using a Leica6000B fluorescence microscope. The image was adjusted each time to a given type of preparation (exposure time, gain, and binning). For analysis of hCFs, 16 images were taken for each experimental condition using the tile scan method. The obtained images were used to determine the efficiency of phenotypic transitions of hCFs into myofibroblasts. Based on the presence of α-SMA-rich stress fibers in hCFs, the efficiency of the FMT process was determined (% of α-SMA-positive cells in the analyzed population). Fluorimetrical analysis was performed using the ImageJ freeware (NIH, Bethesda, MD, USA). The results are presented as the mean fluorescence intensity of the test protein, relative to DNA fluorescence intensity in all 16 images as described previously [33]. For the analysis of activation of selected transcription factors in the tested cells, the level of fluorescence of the phosphorylated form of SMAD2 (pSMAD2) or SNAI2 measured in the area of the cell nucleus was determined [34]. The length and area of the focal contact sites (FCs) from collected images were measured in ImageJ [35]. The obtained fluorimetry values are presented as relative fluorescence units (RFU).

**Table 1:** List of antibodies used in the study

Antibody	Specification	Assay
**Primary antibodies**		
Anti-OCT4	Monoclonal mouse, IgG, Invitrogen #MA1-104	IF (1:200), WB (1:1000)
Anti-SSEA4	Mouse, IgG3, Invitrogen #A24866	IF (1:100)
Anti-CD133	Monoclonal mouse, IgG1, Thermo Fisher Scientific #MA1-219	IF (1:200), WB (1:1000)
Anti-α-SMA	Monoclonal mouse, Sigma-Aldrich #A2547	IF (1:400), WB (1:1000)
Anti-Vinculin	Monoclonal mouse, Sigma-Aldrich #V9264	IF (1:200), WB (1:1000)
Anti-pSmad2	Polyclonal rabbit, Cell Signaling Technology #3108	IF (1:100)
Anti-Slug2	Polyclonal rabbit, Sigma-Aldrich # PRS3959	IF (1:100)
Anti-CD9	Monoclonal mouse, IgG1, Invitrogen #10626D	WB (1:500)
Anti-CD81	Monoclonal mouse, IgG1, kappa, Invitrogen #MA5-13548	WB (1:200)
Anti-Flotillin1	Polyclonal goat, IgG, Invitrogen #PA5-1853	WB (1:1000)
Anti-Transferrin	Polyclonal rabbit, IgG, Invitrogen #PA3-913	WB (1:1000)
Anti-E-Cadherin	Monoclonal rat, IgG1, kappa, eBioscience #14–3249-82	WB (1:500)
Anti-Syntenin	Polyclonal goat, IgG, Invitrogen #PA5-18595	WB (1:250)
Anti-Calnexin	Polyclonal goat, IgG, Invitrogen #PA5-19169	WB (1:1500)
Anti-COL1A1	Polyclonal rabbit, IgG, ABclonal #A1352	WB (1:1000)
Anti-COL3A1	Monoclonal rabbit, IgG, ABclonal #A0817	WB (1:1000)
Anti-β-tubulin	Monoclonal mouse, IgG2a, Invitrogen #MA5-16308	WB (1:2000)
**Secondary antibodies**		
Anti-mouse, AF 647	Goat IgG, Invitrogen #A-21235	IF (1:1000)
Anti-mouse, AF 488	Goat IgG3, Invitrogen #A24877	IF (1:1000)
Anti-mouse, AF 488	Goat IgG, Invitrogen #A11001	IF (1:1000)
Anti-mouse, HRP	Goat IgG, Invitrogen #31430	WB (1:2000)
Anti-rabbit, HRP	Goat IgG, Invitrogen #31460	WB (1:2000–6000)
Anti-goat, HRP	Rabbit IgG, Invitrogen #R-21459	WB (1:2000)
Anti-rat, HRP	Donkey IgG, Invitrogen #A18739	WB (1:2000)

*IF* immunofluorescence, *WB* Western blot, *AF* Alexa Fluor, *HRP* Horseradish peroxidase

### Confocal microscopy

EV uptake by hCFs as well as localization of pSMAD2 in the cells was made using scanning laser confocal microscopy (Zeiss LSM 900 with Airyscan 2) as described before [36]. To analyze EV uptake by hCFs the cells were stained with a DiO lipid probe (3,3′-dioctadecyloxacarbocyanine, perchlorate; Invitrogen). EVs were stained with the Vybrant DiD cell labeling solution (1,1'–dioctadecyl-3,3,3′,3′-tetramethylindodicarbocyanine, 4-chlorobenzenesulfonate salt; Invitrogen). Briefly, EV-H5 were incubated with 5 µM dye in 1 ml PBS for 30 min at 37°C. Next, EVs were purified by SEC using qEV/70 nm columns and concentrated in 2-ml filter tubes with 10 kDa protein cutoff (Amicon). Purified EVs were added to hCFs for 30 min and imaged. 10 cells were analyzed.

To identify the number of pSMAD2-positive cells, confocal tile images were obtained, composed of 5 × 5 scans. 5 randomly selected areas on the sample for each experimental group were analyzed. Next, to determine the localization of pSMAD2 in the cells, high-resolution Airyscan analysis was performed. First, a 2D image of an entire cell was obtained followed by generating a ‘z-stack’ of the nuclear region. a Total of 10 cells for each experimental group were analyzed.

### Cell stiffness measurement by atomic force microscopy (AFM)

AFM analysis was conducted using a Bioscope Catalyst (Bruker) coupled with an inverted optical microscope AxioObserver Z1 (Zeiss). During the analysis, cells were maintained in culture medium at 37°C. Morphology images as well as elasticity maps of the cells were obtained using PeakForce Tapping mode with PeakForce Capture turned ON. This enabled the acquisition of a force curve in every pixel of an image. For AFM imaging a relatively soft cantilever was used with a nominal tip radius of 20 nm and with an experimentally determined spring constant of 0.68 N/m (Bruker Probes). Nanomechanical analysis of cells was made in force spectroscopy mode, which consisted of measuring force–displacement curves. Prior to cell measurement, the AFM probe was positioned on top of the cell and aligned in the center of the cell by optical microscopy. Once aligned, force–displacement curves from a grid of 5 × 5 points were collected at a rate of 1 Hz. 30 cells for each experimental group were analyzed. A detailed description of the mechanical analysis used in this work can be found elsewhere [37]. In the analysis, a soft cantilever was used with a nominal tip radius of 20 nm and with an experimentally determined spring constant of 0.014 N/m (Bruker Probes). Data analysis of the obtained force–displacement curves from both PeakForce Tapping and force spectroscopy was performed using AtomicJ software [38].

### In vivo studies of heart fibrosis

Animal experiments were approved by the I Local Ethical Committee in Krakow (agreement no. 554/2021). Mice (8-week-old male NOD/SCID strain) were housed in an institutional animal unit (Department of Clinical Immunology, Institute of Pediatrics, Jagiellonian University Medical College, Wielicka 265, 30–663 Krakow, Poland) under conditions: 12 h light cycle, standard rodent chow diet, free access to water, in a sterile environment. Fibrosis was induced by angiotensin II (Ang II; Sigma-Aldrich), which was administered subcutaneously in osmotic pumps (Alzet, Cupertino model 1004) at a dose of 1.4 mg/kg/day, according to a previously published protocol [39]. 14 days after induction of fibrosis, the animals received 4 doses of EVs by retro-orbital injection at intervals of 3–4 days (4 × 10e10 EV particles each time, as measured by NanoSight). Injections were performed on alternate eyes (2 injections per eye) with no sign of ocular injury. EVs from normoxia and hypoxia of 5% O_2_ were applied. To adhere to the 3R principle (Replacement, Reduction, and Refinement), 6 animals per group were used. One animal was excluded from the analysis due to the removal of the osmotic pump. The experiment was terminated 14 or 28 days after induction of fibrosis. Mice were euthanized and hearts were collected for molecular and histopathological analyses.

### Histopathology

The hearts were harvested from the experimental animals and fixed in 10% buffered formalin, dehydrated by incubations in EtOH solutions with increasing EtOH content: 50, 70, 96, and 100%, and then in xylene and liquid paraffine. Subsequently, the tissue samples were embedded in paraffin, cut into 4 µm thick sections, and stained with hematoxylin and eosin (H&E) for morphological analyses, using a previously published protocol [40]. Briefly, sections were deparaffinized, washed in xylene, and then rehydrated in EtOH solutions with increasing content of water. After staining in Mayer’s hematoxylin solution (Merck) and subsequent washing in running tap water, they were stained with 0.5% eosin Y solution with phloxine (Merck) (90 s). Next, the sections were again dehydrated in EtOH solutions and washed in xylene. Finally, the slides were sealed with a Histofluid mounting medium (Paul Marienfeld, Lauda-Königshofen, Germany). Microscopic evaluation of H&E samples was carried out by assessing the presence of inflammation according to a scale: 0 = absent, 1 = infiltration of inflammatory cells around the vessels, 2 = in < 50% of the tissue, 3 = in 50–75% of the tissue, 4 = in > 75% tissue.

Collagens were stained with Sirius red dye as previously described [41, 42]. Rehydrated tissue sections were stained with Sirius red dye in aqueous solution of picric acid (Merck) for 1 h, followed by two consecutive washes in glacial acetic acid (0.5% v/v) in deionized H_2_O. Next, the slides were dehydrated in EtOH solutions, followed by washing in xylene. After the staining sections were sealed with Histofluid mounting medium.

### miRNA sequencing

The sequencing of miRNAs in EVs was performed using next-generation Ion TorrentTM technology. First, total RNA was isolated using the Total Exosome RNA & Protein Isolation Kit (Invitrogen), and ribosomal RNA was then removed using the Low Input RiboMinus Eukaryote System 2 kit (Invitrogen). Prior to library preparation, the miRNA fraction was assessed using the Small RNA 2100 Bioanalyzer Kit (Agilent). Libraries were prepared with the Ion Total RNA-Seq v2 kit (Thermo Fisher Scientific) according to the manufacturer's instructions for preparing small RNA libraries. The sequencing template was generated with the Ion PI ™ Hi-Q ™ OT2 200 kit on the Ion OneTouch ™ 2 system. Libraries were sequenced on the Ion Proton Sequencer using the Ion PI Hi-Q Sequencing 200 and the Ion PI Chips v3 chemistry (Thermo Fisher Scientific). Data analysis was performed in Torrent Suite v5.10.0 and miRNA readings were counted using the SmallRNA plugin. The percentage of miRNAs in EVs was calculated in R and in the Excell software. Target genes identification for selected miRNAs was performed using the TargetScanHuman 8.0 [43], the miRDB [44], and the DIANA mirPath v.3 [45] databases. Functional enrichment for miRNA target genes and miRNA-gene network analyses were performed using the MicroRNA Enrichment Turned Network (Mienturnet) [46].

### Transfection of hCFs with miRNA-302 mimic/inhibitor

To verify miR-302b-3p activity on target genes, hCFs were transfected with the miR-302b-3p inhibitor (#AM17000; Ambion), miR-302b-3p mimic (mirVana #4464066; Invitrogen) or anti-miR miRNA negative control (#AM17010; Ambion). Briefly, 3 × 10e4 cells/well were seeded on a 24-well plate a day before. Transfection was done using 50 nM of either control, miR-302b-3p inhibitor, or miR-302 mimic using Dharmafect 1 (Dharmacon) as a transfection reagent, according to the manufacturer’s instruction. Approximately 16 h after transfection the medium was replaced with fresh medium supplemented with 2% FBS and TGF-β (1 ng/ml). Cells were treated with EV-H5 (2.5 × 10e4 EV particles/cell), where indicated, for 24 h. Then, EVs were removed and cells were cultured for the next 24 h, before harvesting for RNA analysis. Three independent experiments were done.

### Quantitative real-time PCR (qPCR)

Total RNA was extracted from cultured cells using the GeneMATRIX Universal RNA/miRNA Purification Kit (EURx). In the case of EVs, RNA was purified with the Total Exosome RNA and Protein Isolation Kit (Invitrogen). For RNA isolation from mouse heart tissue and from hCFs transfected with miR302b-3p mimics/inhibitor, the Fenozol Plus reagent (A&A Biotechnology) was used. The procedures were done according to instructions provided by the vendors. RNA concentration and purity were determined using the Implen spectrophotometer. Reverse transcription was performed with 1–2 µg RNA and the NG dART RT-PCR Kit (EURx) in a C1000 Touch Thermal Cycler (BioRad). The synthesized cDNA was used to analyze the expression level of selected genes by the real-time qPCR method or stored at – 20°C until use. The reaction mixes contained cDNA, SybrGreen dye (Applied Biosystems/Thermo Fisher Scientific), and specific primer pairs. The reaction was run in the 7500 Fast Real-Time PCR System (Applied Biosystems) under the following conditions: 50°C, 2 min; 95°C, 10 min; and 40 cycles: 95°C, 15 s; 60°C, 1 min. Relative gene expression level was calculated using the ∆∆Ct method. The list of primer sequences is provided in Table 2.

**Table 2:** Primer sequences used for qPCR

Gene name	Gene ID	Forward (5′ – 3′)	Reverse (5′ – 3′)
**Human genes**			
***OCT4**** (Octamer-binding transcription factor 4)*	5460	CCTTCGCAAGCCCTCATTTC	TAGCCAGGTCCGAGGATCAA
***SOX2**** (SRY-box transcription factor 2)*	6657	GGGAAAGTAGTTTGCTGCCTC	CAGGCGAAGAATAATTTGGGGG
***NANOG**** (Nanog homeobox)*	79923	ACCTCAGCTACAAACAGGTGAAG	TTCTGCGTCACACCATTGCT
***B2M**** (beta2-microglobulin)*	567	AATGCGGCATCTTCAAACCT	TGACTTTGTCACAGCCCAAGATA
***ACTA2**** (Actin alpha 2, smooth muscle)*	59	CTGTTCCAGCCATCCTTCAT	CCGTGATCTCCTTCTGCATT
***CCN2**** (Cellular communication network factor 2; connective tissue growth factor)*	1490	CCCCAGACACTGGTTTGAAGA	CCTCCCACTGCTCCTAAAGC
***COL1A1**** (Collagen type I alpha 1 chain)*	1277	CTTTGCATTCATCTCTCAAACTTAGTTTT	CCCCGCATGGGTCTTCA
***COL3A1**** (Collagen type III alpha 1 chain)*	1281	CTGGTGGTAAAGGCGAAATG	CCAGGAGCACCATTAGCAC
***FN1**** (Fibronectin 1)*	2335	TGTGGTTGCCTTGCACGAT	GCTTGTGGGTGTGACCTGAGT
***TAGLN**** (Taglin; transgelin)*	6876	CGTGGAGATCCCAACTGGTT	AAGGCCAATGACATGCTTTCC
***TNC**** (Tenascin C)*	3371	GGTCCACACCTGGGCATTT	TTGCTGAATCAAACAACAAAACAGA
***COL1A2**** (Collagen type I alpha 2 chain)*	1278	TGCTGCTGGTCAACCTGGTGC	ACTTCCAGCAGGACCGGGGG
***COL4A1**** (Collagen type IV alpha 1 chain)*	1282	CTAATCACAAACTGAATGACTTGACTTCA	AAATGGCCCGAATGTGCTTA
***TGFB1**** (Transforming growth factor beta 1)*	7040	AGGGCTACCATGCCAACTTCT	CCGGGTTATGCTGGTTGTACA
***SNAI1**** (Snail family transcriptional repressor 1)*	6615	GCTGCAGGACTCTAATCCAGA	ATCTCCGGAGGTGGGATG
***SNAI2**** (Snail family transcriptional repressor 2)*	6591	TGGTTGCTTCAAGGACACAT	GTTGCAGTGAGGGCAAGAA
***PFN1**** (Profilin 1)*	5216	ACCGCCTTCTGGTAATCTTGAG	TCCAGCATCCAGCAGACAAG
***PXN**** (Paxilin)*	5829	CCCTGACGAAAGAGAAGCCTAAG	AGATGCGTGTCTGCTGTTGG
***TLN1**** (Talin 1)*	7094	CCCTGATGTGCGGCTTCG	TGTCCTGTCAACTGCTGCTTC
***SMAD2**** (SMAD family member 2)*	4087	CGTCCATCTTGCCATTCACG	CTCAAGCTCATCTAATCGTCCTG
***TGFBR2**** (Transforming growth factor beta receptor 2)*	7048	GACATCAATCTGAAGCATGAGAACA	GGCGGTGATCAGCCAGTATT
***BMPR2**** (Bone morphogenetic protein receptor 2)*	659	CTCAGTCCACCTCATTCATTTAACCG	ACAGAGACTGATGCCAAAGCAAT
***ROCK2**** (Rho-associated coiled-coil containing protein kinase 2)*	9475	CAACTGTGAGGCTTGTATGAAG	TGCAAGGTGCTATAATCTCCTC
***PFN2**** (Profilin 2)*	5217	GAGACTCTGGGTTCTAGCTGC	ACACCTTTCCCCACCAACAG
***CFL2**** (Cofilin 2)*	1073	GACTCCTTCGCTGTATCGTCT	TCTCTTTTTGATCTCCTCTTGTGT
***PTEN**** (Phosphatase and tensin homolog)*	5728	CTCAGCCGTTACCTGTGTGT	AGGTTTCCTCTGGTCCTGGT
***CD44**** (CD44 molecule)*	960	CAGCTCATACCAGCCATCCAA	GACTGGAGTCCATATCCATCCTT
***CCND1**** (Cyclin D1)*	595	ATGCCAACCTCCTCAACGAC	TCTGTTCCTCGCAGACCTCC
***CCND2**** (Cyclin D2)*	894	GAAGCTGTCTCTGATCCGCA	TGCTCCCACACTTCCAGTTG
***18S rRNA**** (18S ribosomal RNA)*	106631781	GTAACCCGTTGAACCCCATT	CCATCCAATCGGTAGTAGCG
***GAPDH**** (glyceraldehyde-3-phosphate dehydrogenase)*	2597	GAAGGTCGGAGTCAACGGAT	AGTTGAGGTCAATGAAGGGGTC
**Mouse genes**			
***Acta2**** (Actin alpha 2, smooth muscle)*	11475	CAGGTGATCACCATTGGAAACGAACG	GACAGGACGTTGTTAGCATAGAGATCC
***Col1a1**** (Collagen type I alpha 1 chain)*	12842	GGAGAGAGCATGACCGATG	AAGTTCCGGTGTGACTCGTG
***Col3a1**** (Collagen type III alpha 1 chain)*	12825	TGACTGTCCCACGTAAGCAC	GAGGGCCATAGCTGAACTGA
***Ctgf**** (Connective tissue growth factor)*	14219	AGACCTGTGCCTGCCATTAC	ACGCCATGTCTCCGTACATC
***Il6**** (Interleukin 6)*	16193	AGTCCTTCCTACCCCAATTTCC	TGGTCTTGGTCCTTAGCCAC
***Tnfα**** (Tumor necrosis factor alpha)*	21926	CATCTTCTCAAAATTCGAGTGACAA	TGGGAGTAGACAAGGTACAACCC
***Eef2**** (Eukaryotic translation elongation factor 2)*	13629	ACAATCAAATCCACCGCCATC	AGCCATCCTTGCTCTGCTTA

### Western blot analysis

Cells and heart tissue samples were lysed with RIPA lysis buffer (Sigma) supplemented with protease and phosphatase inhibitors (Thermo Fisher Scientific) and sonicated (3 × 10 s). EVs were lysed by adding 1/5 volume of RIPA buffer. The lysates were centrifuged at 4 °C for 10 min at 10,000 *g* to isolate the protein fraction. The protein concentration was measured using a BCA assay (Thermo Fisher Scientific), according to the manufacturer’s instructions. 20 or 4 μg protein samples from cell lysates or EVs (respectively), or 20 µl out of 500 µl of each EV fraction collected from qEV/70 nm column, were separated by SDS-PAGE and then transferred onto polyvinylidene fluoride membranes (PVDF; BioRad) using a semi-dry transfer at 25V, 1.3A for 7–10 min (depending on the protein size) in Trans-Blot Turbo RTA Mini PVDF Transfer Kit (BioRad). Membranes were incubated in 5% (w/v) skimmed milk in TBST (0.05 (v/v) Tween 20 in Tris-buffered saline) or 3% BSA in TBST. The membranes were then incubated overnight at 4°C with primary antibodies diluted in 3% BSA in TBST. Next, they were washed with TBST three times and incubated with horseradish peroxidase (HRP)-conjugated secondary antibodies in the same antibody buffer for 50 min at RT. After washing the membranes three times with TBST, the signal was detected with the chemiluminescence HRP substrate (Merck) in the ChemiDoc XRS + (BioRad) imager. Densitometric analysis of the resulting protein bands was performed with the Quantity One (BioRad) software. The list of antibodies is shown in Table 1.

### Statistical analysis

All results are presented as the mean of the measurements with error bars representing the standard deviation (SD). Statistical analysis was performed based on the analysis of the normality of the distribution using the Shapiro–Wilk test. Homogeneity of variances was assessed using the F-test (two groups) or Brown–Forsythe test (three or more groups). Thereafter, the unpaired or paired standard Student’s *t*-test was used for equal variance or the Welch *t*-test for unequal variance. For multiple comparisons, one-way ANOVA was used followed by the Tukey post hoc test, in case if the data showed normal distribution and equal variances or Welch ANOVA with the Dunnett test for unequal variances. If the data were not normally distributed, the non-parametric Kruskal–Wallis test with Dunn’s post hoc test was used to calculate statistical significance. Statistical analysis was performed using GraphPad Prism 8.4.0 software and the differences were considered statistically significant at *p* < 0.05. Statistical tests used for data analysis are indicated in each figure legend.

## Results

### hiPSCs express higher levels of pluripotency genes *OCT4* and *NANOG* in hypoxia

**Fig. 1:**
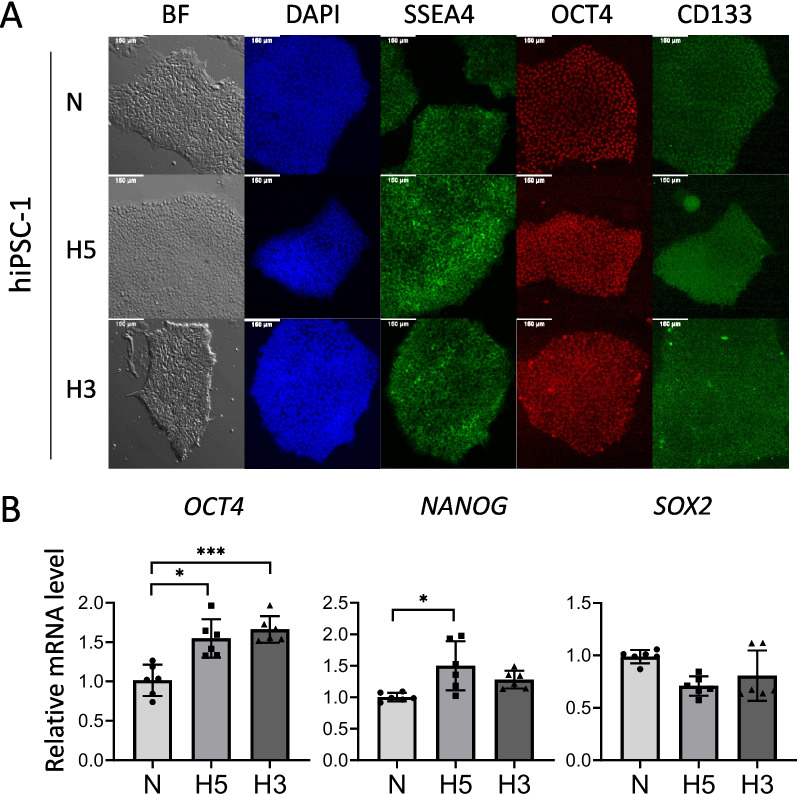
Characteristics of human induced pluripotent stem cells (hiPSCs) cultured in different oxygen conditions. **A** Microscopic images of hiPSCs colonies in bright field (BF) and immunofluorescent staining for the presence of pluripotency markers: SSEA4, OCT4, and CD133. Staining of cell nuclei — DAPI. **B** Analysis of expression levels of transcription factors related to pluripotency: *OCT4*, *NANOG*, and *SOX2* by real-time qPCR (*n* = 6). Cell culture conditions: N — atmospheric oxygen concentration — normoxia; H5 — hypoxia 5% O_2_; H3 — hypoxia 3% O_2_. All data are presented as the mean ± SD. Statistical significance was tested using Welch ANOVA followed by a post hoc analysis with the Dunnett test (**B**). *p < 0.05; ***p < 0.001

In order to explore the regenerative properties of hiPS-EVs derived from hypoxia, we used a hypoxic chamber designed to maintain stable conditions at a defined oxygen level. It is known that the natural niches of pluripotent embryonic stem cells (ESCs) contain 3–5% oxygen [47]. Since iPSCs are considered artificially generated equivalents of ESCs, we selected two oxygen concentrations of 3% (hypoxia; H3) and 5% (hypoxia; H5) in an attempt to model their physiological environment in our experiments. Control cells were cultured at atmospheric oxygen concentration (21% O_2_, normoxia; N) in a standard cell incubator. We used 3 hiPSC lines that were adapted to different oxygen conditions to generate isogenic cell lines. Before collecting conditioned media for EV isolation, the cells were tested for the expression of pluripotency markers. Our results show that hiPSCs expressed high levels of surface molecules SSEA4 and CD133; they also exhibited the expression of OCT4 transcription factor (Fig. 1A and Additional file 1: Fig. S1). Relative quantification of transcript levels revealed that hypoxia at 5 and 3% O_2_ increased the expression of a master regulator of pluripotency — *OCT4*; however, *NANOG* was elevated only at hypoxia H5 (Fig. 1B). The expression of *SOX2* remained at a similar level in all tested conditions (Fig. 1B).

### Isolation and characterization of hiPS-EVs derived from different oxygen conditions

First, we optimized the protocol for the isolation of hiPS-EVs. Two methods were compared: ultrafiltration (UF) combined with size-exclusion chromatography (SEC; UF + SEC) and the “gold standard” method based on ultracentrifugation (UC). A recent report indicated that isolation by UF + SEC resulted in EVs with increased biological functionality compared to the UC method [48]. Since our aim was to obtain highly potent EVs, suitable for testing in a pre-clinical model of heart fibrosis, we used the observation made in the above report as an important guideline for our study. We used only EV-H5 for optimization, and then the protocol was applied to other types of EVs. With respect to the SEC method, the qEV/70 nm columns were utilized. We confirmed that fractions 0.5–1.5 ml contain the most EV particles and the least contaminating proteins (Additional file 1: Fig. S2A, B). Transferrin was used as an indicator of protein contamination, since it is abundantly present in the Essential8 medium that we used to culture hiPSCs. In contrast, EV-associated proteins, including syntenin and flotillin1, as well as pluripotency-related marker CD133, were mostly present in the fractions 0.5–1.5 ml. These fractions were collected for further experiments. Importantly, a sample obtained from Essential8 medium only (processed with the 10 kDa-UF + SEC method) contained almost no proteins or particles (Additional file 1: Fig. S2A), demonstrating the suitability of this medium for EV isolation. Next, we compared the efficiency of EV isolation using membranes for ultrafiltration with different pore sizes, with protein cut-offs at 10, 50, and 100 kDa. The obtained EV fractions were thoroughly characterized using appropriate methods according to the guidelines of the International Society for Extracellular Vesicles (ISEV) [49].

The particle size and concentration of the EVs were measured with the NanoSight instrument (Additional file 1: Fig. S2C, D). The results obtained for 5 different EV batches indicated that there were no significant differences in the size of EVs between the compared samples (Additional file 1: Fig. S2D). The mean EV diameter was approximately 214 ± 9 nm, as measured with the set parameters. Regarding the number of EV particles obtained per ml of conditioned medium, the most efficient was the UF + SEC-based isolation method using filtration tubes with a protein cutoff at 10 kDa (UF-10 kDa + SEC) (Additional file 1: Fig. S2E). Similarly, harvesting EVs by means of the UF-10 kDa + SEC method resulted in the highest protein yield, as measured with BCA (Additional file 1: Fig. S2F). The ratio of particle number to protein concentration was similar in all tested EV groups (Additional file 1: Fig. S2G). The EVs showed the presence of surface markers typical for exosomes (CD9, CD81) and shedding vesicles (flotillin1), as well as the presence of proteins associated with pluripotency (OCT4, CD133, E-cadherin), regardless of the isolation method (Additional file 1: Fig. S2H). The visualization of the EVs with TEM confirmed their circular structure and small size (Additional file 1: Fig. S2I). Considering the highest EV yield obtained using UF-10 kDa + SEC, this method was selected for comparison of EV functionality with the UC method, in the in vitro model of heart fibrosis. To do so, hCFs were stimulated with TGFβ (1 ng/ml) to induce a fibrotic phenotype, characterized by increased expression of pro-fibrotic genes — *ACTA2* and *CCN2*; and subsequently were treated with EVs isolated by the two different protocols. The addition of EVs isolated by the UF-10kDa + SEC method resulted in a more prominent reduction in *ACTA2* and *CCN2* transcript levels, compared to EVs obtained by the UC method (Additional file 1: Fig. S2J). Given the above, EVs isolated using the UF-10kDa + SEC method were selected for further studies.

**Fig. 2:**
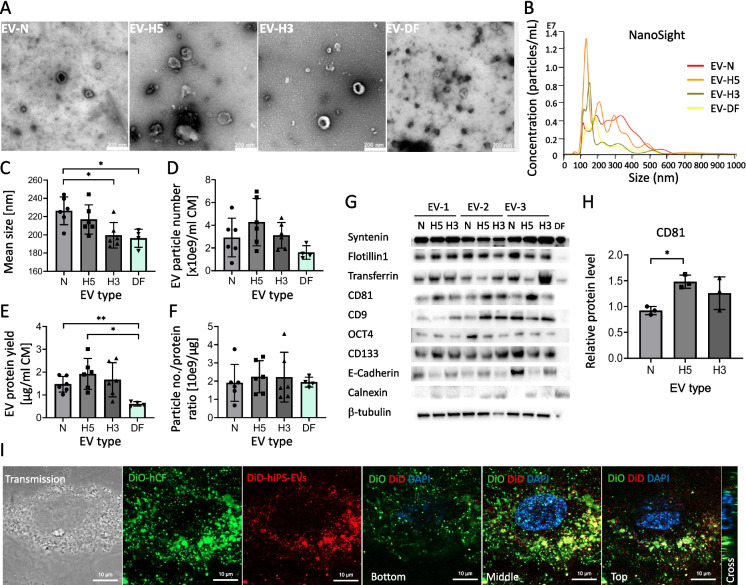
Characteristics of EVs derived from iPSCs cultured under different oxygen conditions: normoxia — 21% O_2_ (EV-N), hypoxia 5% O_2_ (EV-H5), and hypoxia 3% O_2_ (EV-H3). EVs from human dermal fibroblasts (DF-EVs) were used as control. **A** Images of EVs by transmission electron microscopy. **B** Representative histograms of EV size and concentration measured with the NanoSight device. **C** Size analysis of EVs by NanoSight (*n* = 4 for DF-EVs; *n* = 6 for hiPS-EVs). **D** Analysis of EV particle number per ml of conditioned medium (CM) (*n* = 4–6). **E** Analysis of the EV protein yield calculated per ml of CM (*n* = 4–6). **F** Ratio of EV particle number to protein concentration (*n* = 4–6). **G** Western blot analysis of proteins typical for EVs: syntenin, flotillin1, CD9, CD81, a protein present in the cell culture medium (transferrin), pluripotency markers (OCT4, CD133, E-cadherin), endoplasmic reticulum protein (calnexin; a negative marker), and control — β-tubulin. **H** Densitometric analysis of CD81 protein level in EV preps derived from different oxygen conditions (*n* = 3). **I** Confocal microscopy images of DiD-labeled EVs (red) uptake by human cardiac fibroblasts (hCF; stained in green with DiO). Representative images of different cell depths are shown. All data are presented as the mean ± SD. Statistical significance was tested using Welch ANOVA and the Dunnett post hoc test (**C**–**E**) and with the one-way ANOVA and the Tukey post hoc test (**F**, **H**). *p < 0.05, **p < 0.01

Following the optimized EV isolation protocol, we obtained EVs from hiPSCs cultured under different oxygen conditions: 21% O_2_ (normoxia; EV-N) and physiological hypoxia at 5% O_2_ (EV-H5) and 3% O_2_ (EV-H3). In addition, we isolated EVs from biologically neutral cells — dermal fibroblasts (EV-DF) — which served as a control. As previously, all EVs were characterized in detail using appropriate methods: they were visualized by TEM (Fig. 2A), and the size and concentration of EVs were measured with the NanoSight device (Fig. 2B). On average, EVs derived from hypoxic conditions had a smaller diameter compared to those isolated from normoxia (respective mean values: 226 ± 15 nm for EV-N; 217 ± 16 nm for EV-H5 and 200 ± 15 nm for EV-H3; Fig. 2C). Comparison of the EV yield per 1 ml of CM revealed the highest numbers of EV particles obtained from the H5 hypoxic condition, although due to the variability this was not statistically significant (Fig. 2D). The highest protein yield was obtained for EV-H5 (1.9 ± 0.7), followed by EV-H3 (1.7 ± 0.8), and EV-N (1.5 ± 0.3) (Fig. 2E). EV-H5 and EV-N preparations contained significantly higher amount of proteins than EV-DF (0.6 ± 0.1). The ratio of EV particles per 1 µg of protein, which is an indicator of EV purity, was similar in all types of EVs (Fig. 2F). EVs from all oxygen concentrations showed the presence of typical markers for EVs (CD81, CD9, syntenin, flotillin1), factors related to pluripotency (OCT4, CD133, E-cadherin) and transferrin, with syntenin detected in the highest abundance across the samples. At the same time, the EVs did not contain calnexin — a protein associated with the endoplasmic reticulum (Fig. 2G). In particular, CD81 protein levels were markedly elevated in EV-H5 compared to EV-N (Fig. 2H). Importantly, the isolated EVs could be easily internalized by hCFs. To visualize the uptake of EVs by hCFs, DiD-labeled EVs were co-incubated with DiO-labeled hCFs for 30 min and imaged with confocal microscopy. The results show that the red fluorescence of DiD colocalized with the green signal from DiO, indicating the abundant presence of EVs inside hCFs in different cross-sections (Fig. 2I).

### EV-H5 reduce pro-fibrotic markers in activated hCFs more effectively than EV-N or EV-H3

**Fig. 3:**
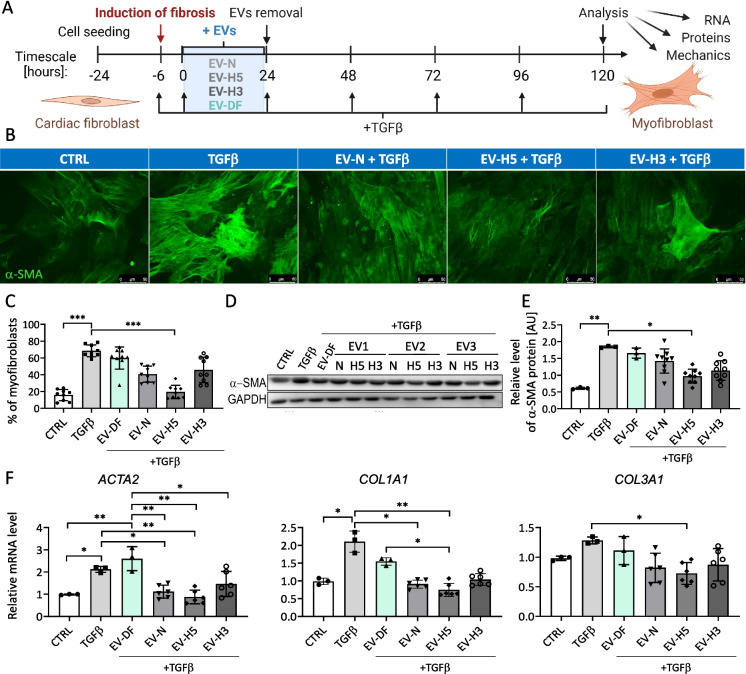
Comparison of the anti-fibrotic effect of hiPS-EVs derived from different oxygen conditions on human cardiac fibroblasts (hCFs). **A** Schematic illustration of the experimental pipeline. **B** Fluorescence-based microscopic analysis of the efficiency of fibroblasts-to-myofibroblasts transition (FMT) in hCFs. α-SMA-positive fibers stained in green. Representative images are shown. **C** Analysis of the percentage of myofibroblasts in hCF populations (% ± SD, *n* = 9). **D** Detection of α-SMA protein in hCFs by Western blot in relation to GAPDH level. **E** Densitometric analysis of α-SMA protein level in hCFs in arbitrary units [AU] (*n* = 3–9). **F** Measurement of the expression level of pro-fibrotic genes (*ACTA2*, *COL1A1*, *COL3A1*) by real-time qPCR method (*n* = 3–6). Abbreviations: CTRL, control hCFs, not treated with TGFβ; TGFβ, hCFs treated with 1 ng/ml of TGFβ; EV-DF, hCFs treated with EVs released by human dermal fibroblasts (hDFs); EV-N, hCFs treated with EVs collected in normoxia (21% O_2_); EV-H5, hCFs treated with EVs collected in hypoxia 5% O_2_; EV-H3, hCFs treated with EVs collected in hypoxia 3% O_2_. All data are presented as the mean ± SD. Statistical significance was tested using the Kruskal–Wallis test with Dunn’s post hoc test (**C**, **E**, **F**: *COL1A1*) and one-way ANOVA and Tukey’s post hoc test (**F**: *ACTA2*, *COL3A1*). *p < 0.05; **p < 0.01; ***p < 0.001; ****p < 0.0001

The main goal of this study was to compare the anti-fibrotic activity of hiPS-EVs generated under different oxygen levels. For this purpose, we isolated EVs obtained in hypoxic environments, and we tested their biological activity on hCFs (Fig. 3A). First, to determine the optimal therapeutic dose of EVs, we treated hCFs with TGFβ (1 ng/ml) and with increasing concentrations of EVs (1.25, 2.5, 5 × 10e4 particles/cell). The expression level of genes characteristic for the pro-fibrotic phenotype of activated fibroblasts (*ACTA2* and *COL1A1*) was analyzed by qPCR. Treatment with EVs resulted in marked but similar reductions in the *ACTA2* transcript levels for all EV concentrations used (Additional file 1: Fig. S3A, left). In contrast, the inhibitory effect on *COL1A1* gene expression was clearly dose-dependent: the dose 1.25 × 10e4/cell decreased *COL1A1* to control levels (by 2.2-fold), and the most prominent reduction (5.4-fold), even below the levels seen in control cells, was attained in response to the highest concentration of EVs (5 × 10e4/cell) (Additional file 1: Fig. S3A, right). The lowest concentration of EVs (1.25 × 10e4/cell) reduced *COL1A1* expression in hCFs by a factor of 1.5 (Additional file 1: Fig. S3A, right). Since the middle dose of EVs (2.5 × 10e4/cell), both had a marked effect on *ACTA2* expression and reduced *COL1A1* to the baseline values, we selected this dose for further studies. The impact of the lowest dose of EVs on *COL1A1* expression was relatively weak, and the highest EV dose could potentially produce a saturation effect when comparing EVs from different oxygen conditions.

Having selected the EV dose, we analyzed the effect of various types of EVs on the metabolic activity of hCFs. Our data show that all hiPS-EVs, from hypoxia and normoxia, transiently enhanced the metabolism of hCFs 24 h after addition (Additional file 1: Fig. S3B, left). However, the effect was statistically significant only for EV-N and disappeared at a later time-point, that is 48 h after treatment (Additional file 1: Fig. S3B, right). DF-EVs remained inert.

Next, we tested EVs from various oxygen conditions in an in vitro model of heart fibrosis (Fig. 3A). Considering that the major trigger of the fibrotic process is the phenotypic transition of fibroblasts to myofibroblasts [9, 50], we analyzed the magnitude of this process in TGFβ-activated hCFs treated with hiPS-EVs. The stress fibers composed of α-SMA protein were imaged by fluorescence microscopy (Fig. 3B) and the numbers of cells with distinct filaments were calculated. The results show that hCFs undergo the phenotypic transition upon treatment with TGFβ, acquiring features typical for myofibroblasts (70 ± 12% compared to 27.7 ± 1.2% in control; Fig. 3C). Although the addition of control EVs isolated from hDFs, as well as EV-N or EV-H3 slightly reduced the percentage of myofibroblasts (to the values of 50.9 ± 7.9%; 45.5 ± 7.2%; 45.1 ± 6.4%, respectively), this effect was not statistically significant. Only EV-H5 treatment caused a statistically significant inhibition of the FMT (to the value of 21.3 ± 8.4%), comparable to the control levels (Fig. 3C). This was also confirmed by analyses of α-SMA protein by Western blot (Fig. 3D, E) and at the relative transcript level by real-time qPCR (Fig. 3F). Furthermore, there was a statistically significant decrease in the expression of the *COL1A1* and *COL3A1* genes in TGFβ-activated hCFs after EV-H5 treatment (Fig. 3F). Given the consistent effect of EV-H5, these EVs were selected for further experimental work.

### EV-H5 impact on mechanical properties of activated hCFs

**Fig. 4:**
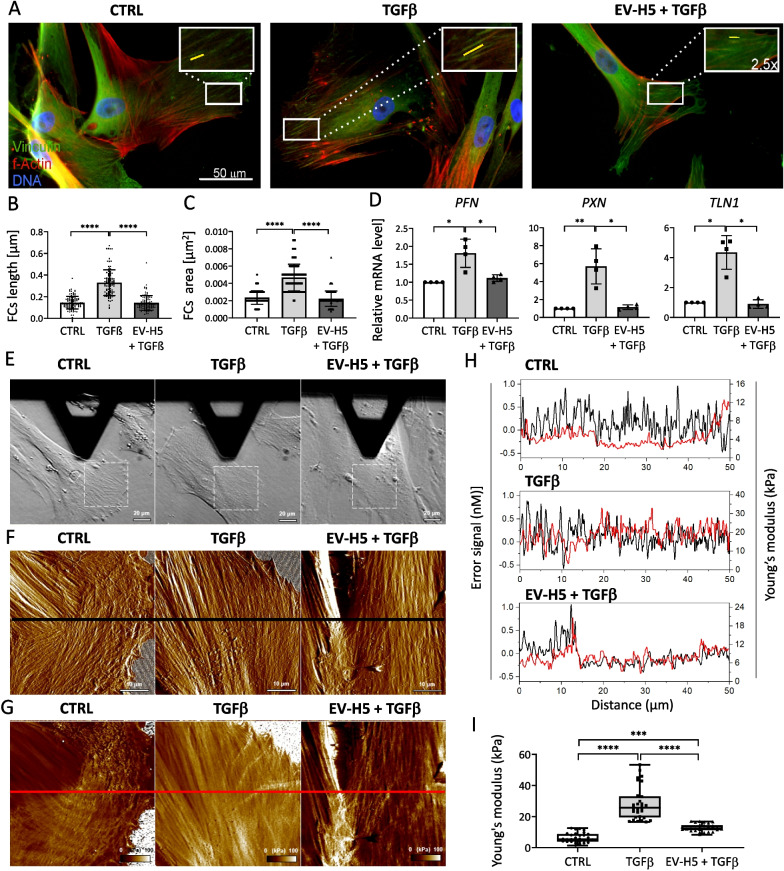
Analysis of hiPS-EV-H5 impact on the actin cytoskeleton, focal contacts, and mechanical properties of human cardiac fibroblasts (hCFs) stimulated with TGFβ (1 ng/ml). EV-H5 from hiPSC-L3 were used. **A** Representative fluorescence microscopy images of hCFs. The insets in the upper corners of the images are zoomed areas marked with white rectangles showing mature focal contacts (FCs). Quantitative data of FC measurements are shown in the graphs below: FCs length (**B**) and area (**C**) (*n* = 105).** D** Analysis of transcript levels of genes associated with the formation of FCs (*PFN, PXN, TLN1*) by real-time qPCR (*n* = 4).** E** Optical microscopy images of cells during AFM analysis, followed by high-resolution morphology images (**F**) as well as elasticity maps (**G**) of the cells. Dash squares in the optical images indicate scanning areas covered by AFM. Black and red horizontal lines in (**F**) and (**G**) indicate cross-sections and the corresponding cross-section analyses are shown in (**H**). **I** Scatter graph showing values of the Young’s modulus determined with AFM force spectroscopy (*n* = 30). All data are presented as the mean ± SD. Statistical significance was tested using Kruskal–Wallis with Dunn’s post hoc test (**B**–**D**, **I**). *p < 0.05; **p < 0.01; ***p < 0.001; ****p < 0.0001

Knowing that myofibroblasts are characterized by contractile capacity due to the presence of α-SMA-rich stress fibers and so-called super-mature focal contacts allowing the generation of appropriate mechanical forces, we investigated the effect of EV-H5 on the structural and mechanical properties of activated hCFs. For this purpose, we analyzed the focal adhesion sites in hCFs activated with TGFβ and treated with EV-H5. Fluorescence images indicated that TGFβ-activated cells developed thick actin stress fibers and large focal contacts rich in vinculin (Fig. 4A–C). Importantly, the obtained results were similar for EV-H5 derived from two other hiPSC lines (Additional file 1: Fig. S4A–C). The length and surface area of focal contacts were significantly decreased in cells exposed to therapeutic EVs (Fig. 4A–C, Additional file 1: Fig. S4A–C). Furthermore, the expression level of genes encoding proteins related to the structure and function of focal contacts, i.e., profilin (*PFN*), paxillin (*PXN*), and talin 1 (*TLN*), was significantly reduced in TGFβ-activated hCFs treated with EV-H5 (Fig. 4D, Additional file 1: Fig. S4D).

Next, we employed AFM to obtain high-resolution images of both the morphology and elastic properties of the investigated cells [51]. Peak force error images revealed that hCFs activated with TGFβ were more flattened and rigid compared to control cells and cells treated with EV-H5 (Fig. 4E). Furthermore, the elasticity maps indicated that cells activated with TGFβ had much higher values of Young’s modulus compared to control cells and cells treated with EV-H5 (Fig. 4F–I). In particular, high values of the Young’s modulus coincided with the extension of actin stress fibers indicating that the actin cytoskeleton was primarily responsible for the cell nanomechanical properties as shown in the cross-section graphs of the cells (Fig. 4H). To quantify the values of Young’s modulus, we employed AFM force spectroscopy measurements [52]. The mean values of the Young’s modulus increased in activated hCFs from 6.36 ± 3.05 kPa, as measured for control cells, to 28.22 ± 10.45 kPa, and were significantly reduced upon treatment with EV-H5: 12.78 ± 2.31 kPa (Fig. 4I). These results indicate that the size of focal adhesions determines the thickness and tension of the actin stress fibers, which determines cell elasticity [53].

### EV-H5 affect SMAD2 signaling leading to the reduction of fibrosis

**Fig. 5:**
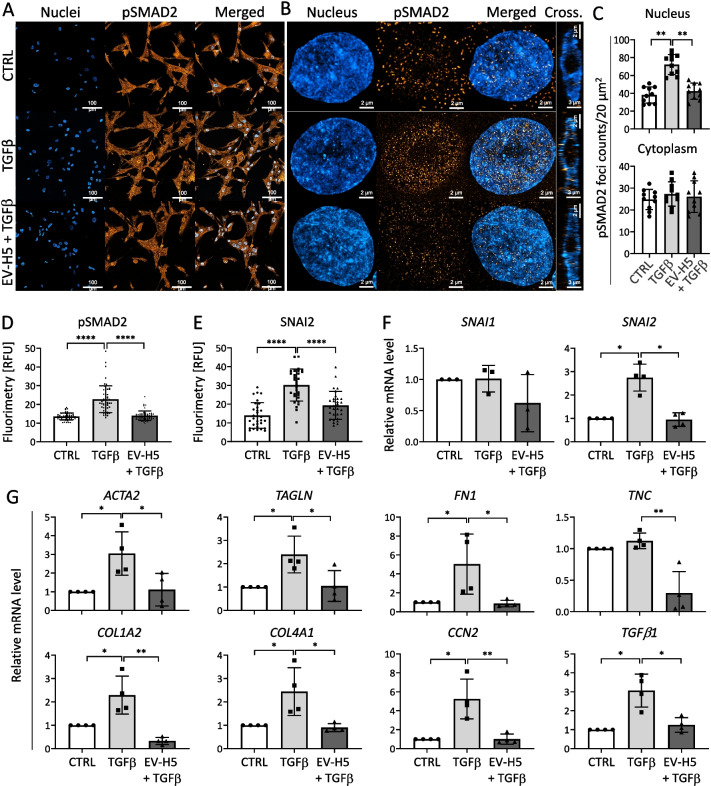
Analysis of fibrosis-related signaling pathway involving SMAD2 and SNAI2 transcription factors in human cardiac fibroblasts (hCFs) stimulated with TGF-β (1 ng/ml) and treated with EV-H5 (from hiPS-L3). **A** Confocal microscopy tile scans containing 5 × 5 images of cells, followed by high magnification Airyscan images of individual nuclei (**B**). **C** Quantification of pSMAD2 foci in the nucleus (upper panel) or cytoplasm (lower panel) in hCFs (*n* = 10). Fluorimetric analysis of pSMAD2 (*n* = 55) (**D**) and SNAI2 (*n* = 30) (**E**) protein level in cell nuclei. **F** Relative quantification of *SNAI1* (*n* = 3) and *SNAI2* (*n* = 4) genes expression in the tested samples by real-time qPCR. **G** Analysis of mRNA levels of pro-fibrotic genes in activated hCFs after EV-H5 treatment (*n* = 4). All data are presented as the mean ± SD. Statistical significance was tested using Welch ANOVA and Dunnet’s test (C) or with Kruskal–Wallis with Dunn’s post hoc test (D-G). *p < 0.05; **p < 0.01; ***p < 0.001; ****p < 0.0001

To decipher the molecular mechanisms underlying the functional effect triggered by EV-H5, we focused on the canonical TGFβ/SMAD2 signaling pathway in hCFs. For this purpose, we analyzed the intracellular localization and phosphorylation of SMAD2, a protein regulated by TGFβ that controls the expression of pro-fibrotic genes [54]. Fluorescent images showed an enrichment of the phosphorylated form of the SMAD2 protein in the nuclei of hCFs activated with TGFβ (Fig. 5A, B), which was confirmed by quantification of pSMAD2 foci (Fig. 5C) and fluorimetry (Fig. 5D, Additional file 1: Fig. S5A, C). Exposure of the cells to therapeutic EV-H5 resulted in a significant decrease in intracellular localization of the pSMAD2 protein in the cell nucleus (Fig. 5A–D, Additional file 1: Fig. S5A, C).

The activity of other transcription factors involved in FMT, such as SNAI1 and SNAI2, was also analyzed. Literature reports indicated the potential role of SNAI1 in the phenotypic transitions of hCFs to myofibroblasts [55]. Our results revealed that in TGFβ-activated hCFs, SNAI2 (also known as SLUG), was elevated in the nuclear region. Treatment of TGFβ-activated hCFs with EV-H5 caused a significant reduction of the SNAI2 protein in the nuclear region to the levels seen in control cells (Fig. 5E, Additional file 1: Fig. S5B, D). Gene expression analysis confirmed the downregulation of *SNAI2*, but not *SNAI1*, in hCFs activated with TGFβ and treated with EV-H5 (Fig. 5F, Additional file 1: Fig. S5E).

Since activation of SMAD2 leads to the induction of genes involved in fibrosis, we measured the expression levels of several profibrotic genes in activated hCFs in response to EV-H5 treatment. The results clearly indicate that EV-H5 markedly reduced the increases in the transcript levels of genes encoding cytoskeletal myofibroblast markers, such as *ACTA2* and *TAGLN*, as well as genes related to the extracellular matrix, including *FN1* (fibronectin), *TNC* (tenascin C), collagens (*COL1A2, COL4A1*) (Fig. 5G, Additional file 1: Fig. S5F). Similar observations were made for genes encoding other proteins related to the phenotypic transition of fibroblasts to myofibroblasts, such as *CCN2* (also known as *CTGF*) and *TGFB1* (Fig. 5G, Additional file 1: Fig. S5F).

### EV-H5 are enriched in miR-302b-3p with anti-fibrotic function

**Fig. 6:**
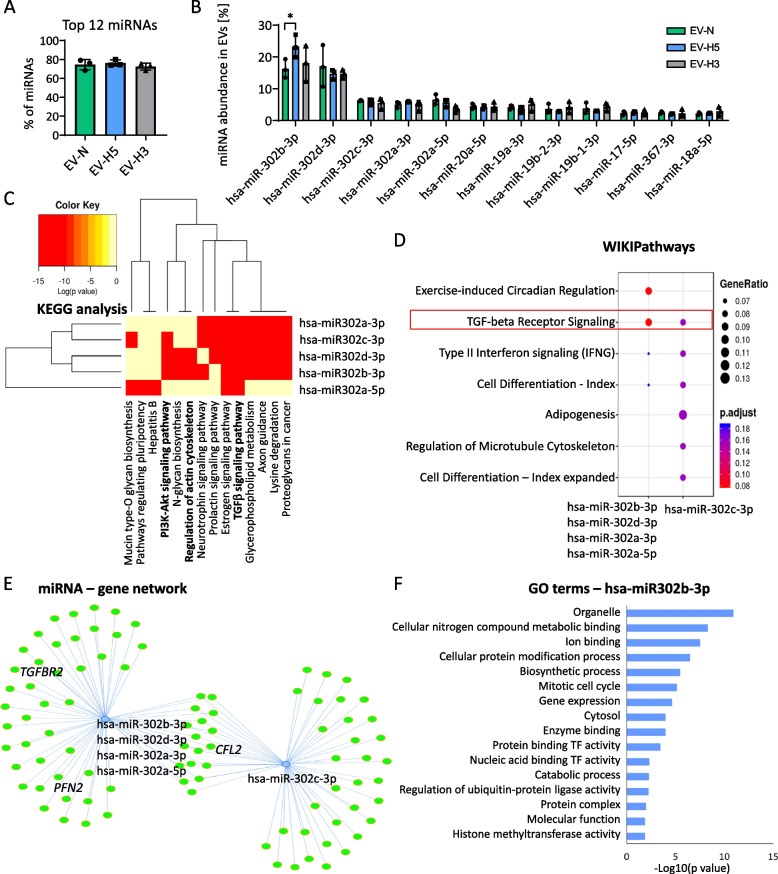
Analysis of the composition and functional networks of miRNAs present in hiPS-EVs. EVs were isolated from 3 iPSC lines cultured in different oxygen conditions: N-normoxia (21% O_2_); H5 — hypoxia 5% O_2_; H3 — hypoxia 3% O_2_. **A** Percentage of the 12 most abundant miRNAs in EVs based on miRNA-seq analysis (*n* = 3). **B** Comparison of the content of individual miRNAs from the Top 12 miRNAs in hiPS-EVs (*n* = 3). **C** Heatmap of KEGG pathways for the miR-302 cluster generated by the mirPath v.3 software (DIANA Tools). WIKIPathways analysis of target genes for miR-302 cluster (**D**) and miRNA-gene network with depicted genes from TGFβ pathway (*TGFBR2*, *PFN*, *CFN*) (**E**) generated by the Mienturnet web tool. **F** Gene ontology (GO) terms (biological process, cellular component, and molecular function) for hsa-miR-302b-3p target genes created with the mirPath v.3 software. TF – transcription factor. All data are presented as the mean ± SD. Statistical significance was calculated in R using *t*-test (**B**). *p < 0.05

Given that miRNAs play an important role in mediating the biological activity of EVs, we investigated the miRNA composition in EVs derived from different oxygen conditions. We carried out high-throughput sequencing followed by bioinformatic analysis to identify the most prominent miRNA species and their possible targets. We focused only on the most abundant miRNAs in EVs, since such miRNAs are most likely to exert biological function in the recipient cells upon delivery. Interestingly, we found that only 12 miRNAs accounted for 74.3 ± 1.9% of the total miRNA content in all EV groups (Fig. 6A). The most abundant miRNAs identified in EVs belonged to the pluripotency-associated miR-302 cluster (miR-302b, miR-302d, miR-302c, miR-302a), with miR-302b-3p present at the highest level (Fig. 6B). Importantly, miR-302b-3p was differentially enriched in EVs depending on oxygen conditions, showing the highest abundance in EV-H5 compared to EVs derived from other oxygen concentrations. The Kyoto Encyclopedia of Genes and Genomes (KEGG) pathways analysis of target genes for miR-302 cluster using DIANA miRpath v.3 web tool pointed towards its role in TGFβ signaling, regulation of actin cytoskeleton and PI3K-Akt signaling (Fig. 6C), all of which are implicated in the development of fibrosis. A remarkable impact on the TGFβ pathway was also confirmed by the WIKIPathways analysis (Fig. 6D) and using the Mienturnet web tool (Fig. 6E). Several target genes for miRNAs from the miR-302 cluster, which may affect the fibrotic pathway (*TGFBR2*, *PFN2*, *CFL2*) are depicted in the graph of the miRNA – gene network (Fig. 6E). The involvement of miR-302b-3p in the regulation of cellular metabolism and gene expression is highlighted in the selection of specific gene ontology (GO) terms, including cellular nitrogen compound metabolic process, ion binding, cellular protein modification process, biosynthetic process, mitotic cell cycle, and gene expression (Fig. 6F).

**Fig. 7:**
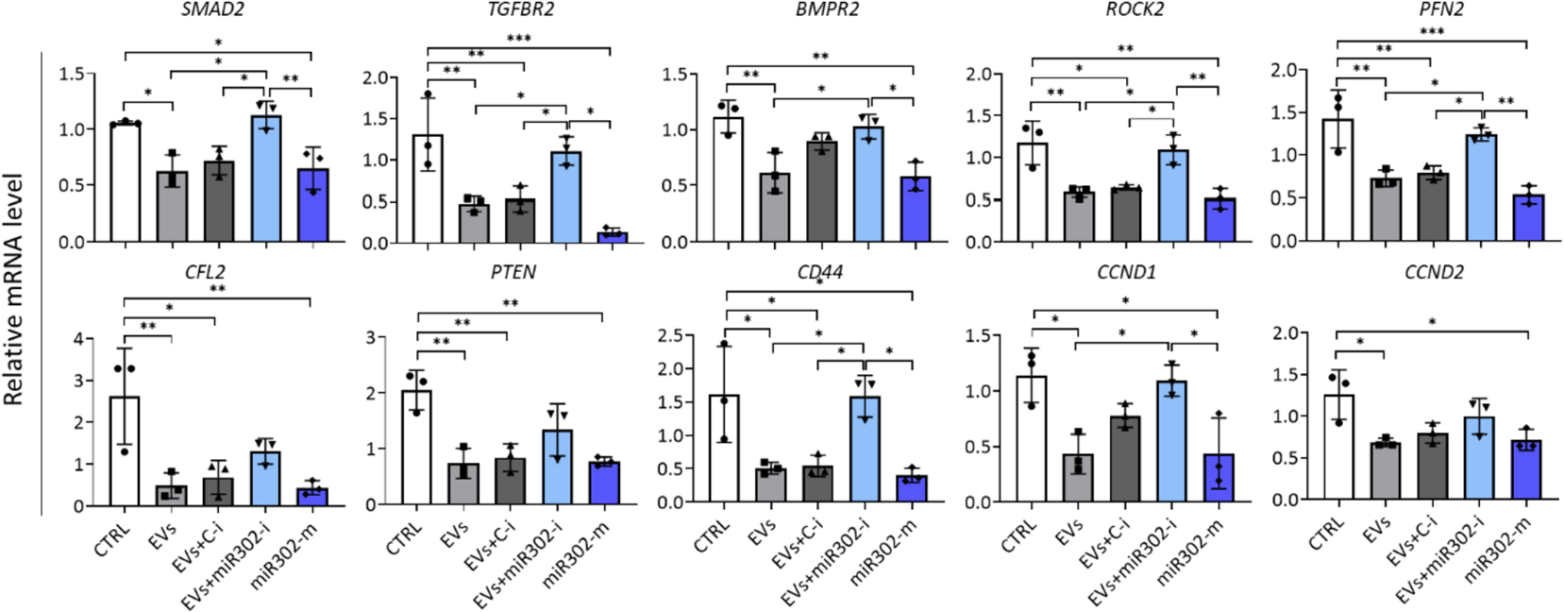
Validation of hsa-miR-302b-3p target genes in human cardiac fibroblasts (hCFs) upon EV-H5 (from hiPS-L3) treatment with the real-time qPCR method. hCFs were transfected with specific miRNA inhibitors or miRNA mimic and treated with EV-H5 and TGFβ (1 ng/ml). Mock-transfected and TGFβ-treated cells were used as control (CTRL). Targets for miR-302b-3p were selected using the TargetScanHuman 8.0, the miRDB, and the DIANA mirPath v.3 databases. Abbreviations: C-i, miRNA-non-targeting inhibitor; miR302-i, miR-302b-3p inhibitor; miR302-m, miR-302b-3p mimic. All data are presented as the mean ± SD (*n* = 3). Statistical significance was tested using one-way ANOVA and the Tukey post hoc test. *p < 0.05; **p < 0.01; ***p < 0.001

To verify the role of miR-302b-3p in the biological function of hCFs with a focus on fibrosis, we selected several potential target genes for this miRNA and analyzed their expression after cell transfection with a specific miRNA inhibitor or mimic. hCFs transfected with control inhibitor and mock-transfected cells were used as controls. The results show a negative regulation of transcript levels for key factors involved in the TGFβ signaling (*SMAD2*, *TGFBR2* and *BMPR2*) and the regulation of actin cytoskeleton (*ROCK2*, *PFN2*, *CFL2*) (Fig. 7). We observed a strong reduction in mRNA levels for these genes after treatment with EV-H5 alone or with a control inhibitor, which was comparable to the effect elicited by the miR-302b-3p mimic. Conversely, the addition of miR-302b-3p inhibitor abolished the effect of EV-H5 in hCFs. A similar response was attained for genes involved in the regulation of cell motility (*PTEN*, *CD44*), and, to some extent, for cell cycle regulators (*CCND1*, *CCND2*) (Fig. 7). These data strongly suggest a crucial role for miR-302b-3p in the anti-fibrotic function of EV-H5.

### EV-H5 reduce inflammation and ameliorate heart fibrosis in vivo

**Fig. 8:**
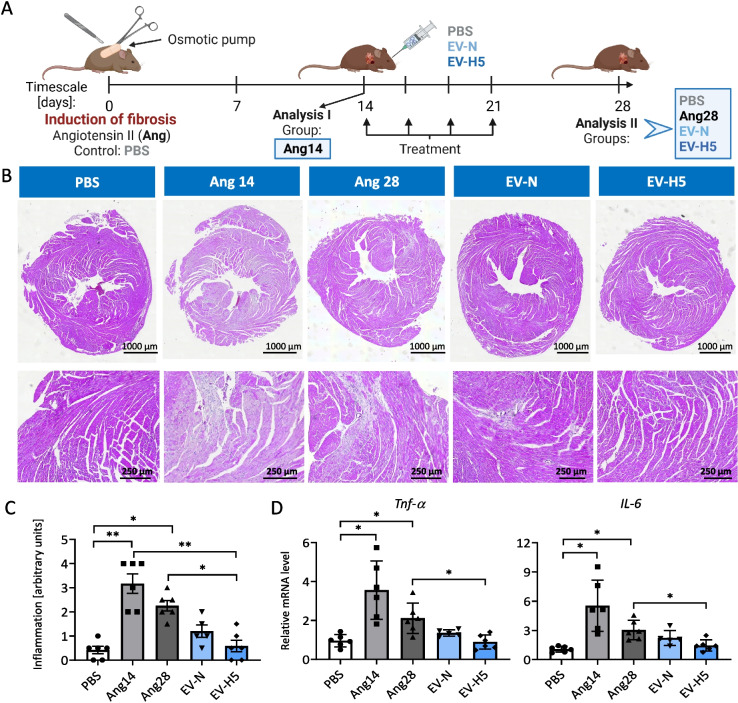
Assessment of the inflammatory status in mouse hearts after treatment with angiotensin II and hiPS-EVs. **A** Schematic illustration of the experimental pipeline. **B** Hematoxylin–eosin staining of heart tissue cross-sections. Inflamed areas with visible infiltration of immune cells can be recognized as bluish areas in contrast to a healthy tissue in pink. Representative pictures of cross-sections (upper panel) and zoomed areas (lower panel) are shown. **C** Relative quantification of immune cell infiltration in the heart tissue (*n* = 5–6). **D** Analysis of transcript levels for pro-inflammatory cytokines (*Tnf-α*, *Il-6*) measured with real-time qPCR method (*n* = 5–6). Abbreviations: PBS, control mice without induction of fibrosis; Ang14, fibrotic mice analyzed 14 days after angiotensin II administration in osmotic pumps; Ang28, fibrotic mice analyzed at the end of the experiment (day 28). Fibrotic mice received EVs from the atmospheric oxygen concentration — EV-N or from the 5% O_2_ hypoxia (EV-H5). All data are presented as the mean ± SD. Statistical significance was tested using Kruskal–Wallis and Dunn’s post hoc test (**C**) and Welch-ANOVA with Dunnett’s post hoc test (**D**). *p < 0.05; **p < 0.01

To test whether the anti-fibrotic effect of EV-H5 found in in vitro studies is also relevant in vivo, we investigated these EVs in a mouse model of heart fibrosis (Fig. 8A). Fibrosis was induced by chronic administration of angiotensin II at a dose of 1.4 mg/kg/day for 28 days, using subcutaneously implanted osmotic pumps. The animals received 4 doses of therapeutic EVs (4 × 10e10 EV particles each time), which were injected every 3–4 days via the retroorbital route, starting on day 14 after induction of fibrosis. We also used EV-N for comparison. Control animals received PBS. The experiment was terminated on day 28 and the mouse hearts were extracted for histopathology and molecular analyses. To monitor the progression of fibrosis, one experimental group was analyzed on day 14 (Ang14), just before the first administration of EVs.

Heart tissue remodeling upon injury or damage is characterized by increased cardiomyocyte death, inflammation, and fibrosis [10]. Therefore, we first evaluated the condition of the hearts from all experimental groups. Heart cross-sections stained with H&E showed immune cell infiltration in groups exposed to angiotensin II, which was most prevalent on day 14 and somewhat diminished on day 28 (Fig. 8B, C). In both EV-treated groups, inflammation was attenuated, although this effect was statistically significant only for EV-H5 (Fig. 8C). This observation was confirmed at the mRNA level of selected proinflammatory cytokines, *Tnf-a* and *Il-6*, whose expression was significantly reduced after EV-H5 treatment (Fig. 8D).

**Fig. 9:**
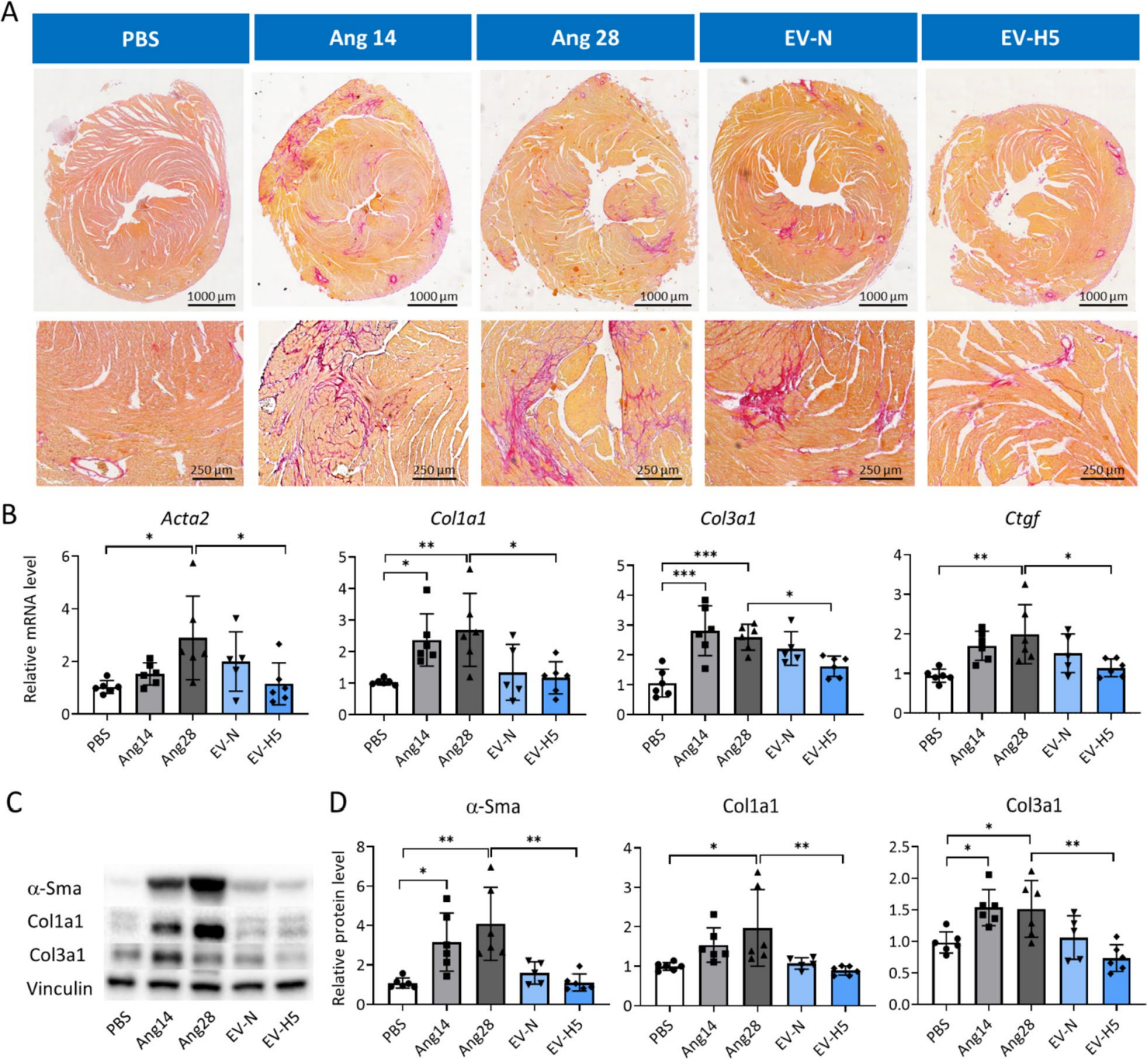
Analysis of fibrosis in mice hearts after treatment with angiotensin II and hiPS-EVs. Mice received angiotensin II for 14 (group Ang14) or 28 days (the rest of the animals) in osmotic pumps implanted subcutaneously. Next, they were treated with hiPS-EVs derived from normoxia (EV-N) or hypoxia 5% O_2_ (EV-H5). Control animals received PBS only (group PBS). **A** Microscopic images of collagen staining in mouse hearts using the Sirius red dye. Cross-sections (upper panel) and zoomed areas (lower panel) are presented. **B** Analysis of transcript levels of key genes involved in fibrosis (*Acta2*, *Col1a1*, *Col3a1*, *Ctgf*), performed by real-time qPCR (*n* = 5–6). **C** Analysis of pro-fibrotic proteins (α-Sma, Col1a1, and Col3a1) by Western blot. **D** Densitometric analysis of the protein levels normalized to the level of control protein (Vinculin) (*n* = 5–6). All data are presented as the mean ± SD. Statistical significance was tested using one-way ANOVA with the Tukey post hoc test (**B**, **D** — Col3a1) or Kruskal–Wallis and Dunn’s post hoc test (**D** — α-Sma, Col1a1). *p < 0.05; **p < 0.01; ***p < 0.001

We next assessed the level of fibrosis in the mouse hearts. The deposition of collagens was analyzed by staining the heart sections with Sirius red dye. Selected representative images show vast areas containing collagen fibers (stained in red) in angiotensin II groups (Fig. 9A). Administration of therapeutic EVs reduced collagen deposition, particularly in the group treated with EV-H5 (Fig. 9A). Relative quantification of mRNA levels for genes involved in the development of fibrosis (*Acta2*, *Col1a1*, *Col3a1*, and *Ctgf*) showed a strong anti-fibrotic effect of EV-H5, which outperformed EV-N (Fig. 9B). Similar observations were made when assessing protein levels for α-Sma, Col1a1, and Col3a1 in the mouse hearts (Fig. 9C). Elevated levels of profibrotic proteins induced by angiotensin II were significantly reduced after EV-H5 treatment (Fig. 9D). These data indicate that EV-H5 exhibit beneficial anti-fibrotic properties both in vitro and in vivo, which could be leveraged for the treatment of myocardial fibrosis.

## Discussion

Efficient targeting of cardiac fibrosis remains a challenge for modern medicine. Although various therapeutic strategies have been tested to restore the physiological balance between ECM production and degradation to prevent heart scarring, none is routinely used in clinical practice [10]. The anti-fibrotic treatment is hampered by the complexity of the molecular pathways implicated in the development of fibrosis which makes it difficult to design a specific drug. Currently, applied treatment strategies rely on the use of inhibitors of the renin–angiotensin–aldosterone system (RAAS), including renin blockers, inhibitors of the angiotensin-converting enzyme, angiotensin receptor blockers, and aldosterone antagonists [56]. Other anti-fibrotic molecules were designed to disrupt the TGFβ signaling pathway [57]. However, paradoxically, the use of pan-TGFβ antibody drugs in cancer patients caused cardiotoxicity [58, 59]. In addition to biomolecules, various stem cell-based therapies were developed in order to reduce fibrotic scar formation and improve heart function [60]. Importantly, the reparative role of stem cell transplants was primarily attributed to the paracrine factors released by these cells, which helped to mitigate the detrimental effects of prolonged inflammation and excessive activation of fibroblasts. Successful suppression of overactive cardiac fibroblasts has also been achieved with cytotoxic T cells modified to express a chimeric antigen receptor (CAR) targeting a myofibroblasts marker — the fibroblast activation protein [61]. Although promising, this treatment strategy is still in its infancy and much research is needed to obtain an effective anti-fibrotic cure.

In our work, we utilized a novel class of therapeutics, based on EVs. Owing to their excellent biophysical properties, EVs can be successfully used as drug delivery tools, with superior characteristics compared to lipid-based nanocarriers [14]. EV-based nanomedicines have already progressed to a pre-clinical and clinical stage, demonstrating efficacy as immunomodulatory, pro-regenerative, and anti-apoptotic agents, as well as vaccines [62]. We took advantage of the exceptional characteristics of EVs and contributed to this rapid development of EV-based bio-active drugs by exploring the functional properties of hiPS-EVs derived from different oxygen conditions, to improve heart function.

The cellular environment has long been recognized as a key factor governing cell identity via direct or indirect mechanisms. Changes in the microenvironment may alter the epigenome of cells and drive evolutional forces [63]. Particularly in the case of pluripotent stem cells, the partial pressure of oxygen has been shown to play a critical role for the maintenance of pluripotency [47]. Low oxygen not only supports the expression of core transcription factors linked to pluripotency, such as OCT4 and NANOG, but also increases genome stability and multilineage differentiation potential [24]. In this work we confirmed that physiological hypoxia (5% O_2_) enhances the expression of *OCT4* and *NANOG* in hiPSCs (Fig. 1). More importantly, we demonstrated that changes in the environment of hiPSCs affect the molecular cargo of the EVs, altering their biological function. Strikingly, hiPS-EVs derived from hypoxic conditions at 5% O_2_ exerted stronger anti-fibrotic activity, compared to EVs derived from normoxia (21% O_2_) or hypoxia 3% O_2_. This observation was consistent for EVs derived from three independent hiPSC lines, which were used in this study. This allows us to conclude that the significant enhancement of the anti-fibrotic function of hiPS-EV in hypoxia is independent of the cell line, despite the differences that naturally occur between the individual hiPSC lines [64].

Other critical factors that affect the cargo of EVs, are the composition of the media for cell culture [65], the storage conditions of EVs [66], and the isolation procedure [67, 68]. In our study, we used a serum-free and chemically defined cell culture medium to propagate hiPSCs, which is also available in a good manufacturing practice grade. Therefore, this cell culture system may easily be adapted to pharmaceutical production in the future. To isolate biologically active EVs, we selected the UF + SEC method with UF tubes with a protein cut-off at 10 kDa, which generated a higher number of EV particles and increased protein yield, compared to UF tubes with a higher protein cut-off (Additional file 1: Fig. S2). EVs isolated this way reduced the expression of pro-fibrotic markers in activated hCFs more efficiently than EVs obtained with the UC method (Additional file 1: Fig. S2J). The increased potency of EVs purified with UF + SEC is consistent with a previous study [48] and may partially be attributed to better homogeneity of the obtained EVs, since the UC method was shown to induce the aggregation of EVs [69]. Moreover, we cannot exclude the possibility of co-purification with EVs of other bioactive molecules, such as ribonucleoproteins, exogenous RNAs, or exomeres [70]. Such molecules may decorate the surface of EVs affecting their biological functions.

Comparison of EVs derived from different oxygen concentrations (N, H5, and H3) revealed a higher number of EV particles obtained for EV-H5, compared to other conditions (Fig. 2D). This observation is consistent with data from other researchers, indicating that low oxygen availability triggers the release of EVs via the hypoxia-inducible factor (HIF)-dependent pathway (reviewed in [26]). We also noted that EVs obtained in hypoxia had a smaller size than EV-N (Fig. 2B, C), which corresponded to a higher level of CD81 protein (Fig. 2G, H). Since CD81 is an established marker for exosomes (small EVs) [49], we conclude, that we have enriched our EV-H5 preparations with a subpopulation of exosomes. However, further research is needed to distinguish individual fractions in the heterogeneous population of EVs, preferably by using single-vesicle technologies [71], and to determine their biological function.

Interestingly, our analysis of the EV-associated protein markers showed that the most abundant expression was detected for syntenin, regardless of oxygen condition and the type of producer cells (Fig. 2G). This protein was easily detected in both hiPS-EVs and DF-EVs. Syntenin was described to be involved in exosome biogenesis via direct interaction with ALG-2-interacting protein X (ALIX) [72] and recently was proposed as a universal biomarker of exosomes [73]. This study appears to support this notion.

The comparison of the antifibrotic properties of hiPS-EVs revealed that all types of EVs reduced fibrotic markers in activated hCFs. However, treatment with EV-H5 led to the most significant reversal of the fibrotic phenotype (Fig. 3). We showed that EV-H5 ameliorated the formation of α-SMA-positive stress fibers and attenuated the length and area of focal adhesion sites, which resulted in reduced cell stiffness (Fig. 4, Additional file 1: Fig. S4). On the molecular level, EV-H5 prevented the translocation of pSMAD2 to the cell nucleus, and downregulated the expression of several profibrotic gene transcripts (Fig. 5, Additional file 1: Fig. S5). Thus, by disrupting the fibrotic pathway in hCFs, EV-H5 prevented their transformation to pathogenic myofibroblasts.

Mechanistically, we identified that the microRNA (miRNA) cargo of hiPS-EVs was primarily responsible for attenuating myofibroblast differentiation of hCFs and the production of fibrotic markers.

Although recent studies quantifying the content of miRNAs per single EV particle revealed their small amount (approximately one copy per 10–100 EVs) [74, 75], the role of miRNAs in the biological activity of EVs exerted on recipient cells has been well documented [12, 76]. In addition, the miRNA regulatory network has been shown to be an important factor in the regulation of cardiovascular homeostasis [77].

Our miRNA screening revealed that the pluripotency-associated miR-302 cluster was the most abundant in hiPS-EVs, which is consistent with our previous study [15] and data from other groups [22, 23]. However, there are some differences in the individual miRNA species detected at the highest level in hiPS-EVs in different studies, which may result from different cell culture systems used. Particularly, we detected a significantly elevated level of miR-302b-3p in EV-H5, compared to EVs released under other oxygen conditions (Fig. 6). Considering the essential role of miR-302 cluster in the maintenance of pluripotency in hiPSCs, we speculate that the higher level of miR-302b-3p in H5-EVs may be related to the fine-tune regulation of pluripotency factors in hiPSCs. EVs in this case may be utilized to egress the excessive amount of unused RNAs, including miRNAs, and other biomolecules. This hypothesis, however, needs further verification and is currently under investigation.

With respect to its function in mitigating fibrosis, our bioinformatics analysis revealed a critical role of miR302b-3p in the regulation of TGFβ pathway and actin cytoskeleton, which are both severely affected in fibrotic disease. To support this statement, we provide direct evidence of a downregulation of transcript levels for several genes from the TGFβ/SMAD pathway, actin cytoskeleton, the regulators of cell motility and cell cycle, after treatment of hCFs with EV-H5 or miR-302b-3p mimic (Fig. 7). This effect was abolished when EV-H5 were used along with the miR302b-3p inhibitor. Our results are in line with and extended data from other reports showing the inhibitory effect of miR-302 family members on TGFβ/SMAD2 signaling [23, 78]. Based on sequence similarity, these miRNAs may cooperate and target a similar set of genes, boosting the biological effect.

The achieved enhancement of the antifibrotic properties of EVs obtained from physiological hypoxia was further confirmed in in vivo studies. The development of fibrotic scar during the natural healing process is always preceded by extensive inflammation [10]. We have shown that EV-H5 inhibit infiltration of immune cells in mouse hearts and reduce the level of pro-inflammatory cytokines (Fig. 8). The resulting decreased level of proinflammatory mediators further translated into reduced deposition of collagens and downregulated expression of α-SMA in the heart tissue (Fig. 9). Our data remain in agreement with other reports, confirming the anti-inflammatory properties of hiPS-EVs [18] and their potential application as anti-fibrotic drugs [22, 23]. We advanced these observations by demonstrating that hiPS-EVs derived from physiological hypoxia exhibit significantly enhanced anti-inflammatory, anti-fibrotic, and pro-regenerative properties. Data presented in this work open new avenues in the therapeutic application of EVs to treat heart fibrosis. However, translating these findings into the clinical practice requires further studies in order to (i) fully optimize the dose and the treatment regimen in human patients; (ii) select the route of EV administration; (iii) analyze the pharmacokinetics of infused EVs; and (iv) determine the safety profile of EVs. If successfully established and approved, EV-based therapeutics can offer a new solution in the treatment of end-stage heart disfunction. Moreover, given the common molecular background of fibrotic diseases, our findings can be extended beyond the field of cardiology and used for the treatment of fibrotic disease in other organs, such as the liver, lungs, kidney, and pancreas, among others. Using in vitro models and studies in small animals, we are currently testing the antifibrotic properties of H5-EVs in various tissues. Thus, our work may contribute to the development of innovative EV-based treatment strategies applicable for a large group of patients.

## Conclusion

We showed an improved anti-fibrotic activity of EVs obtained from iPSCs cultured under conditions of reduced oxygen concentration (5% O_2_), compared to EVs derived from normoxia (21% O_2_) or hypoxia 3% O_2_. The functional effect of the EVs has been validated in an in vitro model of heart fibrosis and further confirmed in experimental animals. Mechanistically, we identified miR-302b-3p as the major driver of the anti-fibrotic function of hiPS-EVs, whose expression was strongly elevated in the condition of hypoxia 5% O_2_. We have demonstrated that by modulating the cellular environment by changing the level of oxygen availability, we can shape not only the properties of the parental cells, but also the molecular cargo of EVs. This could allow to generate EVs with the desired biological functionality, which can be leveraged for the future clinical applications for the treatment of cardiac fibrosis.

## Limitations of the study

Although very promising, this study does not cover all aspects associated with EV-based activities. Considering the complexity of the cellular composition of the heart tissue, EVs may trigger different responses in distinct cell types, in particular cardiomyocytes and endothelial cells, which were not evaluated in this work. Furthermore, we focused only on miRNA cargo in EVs, and we pointed to miR-302b-3p to be primarily responsible for the reduction of heart fibrosis. However, other bioactive molecules, such as proteins, lipids, and other RNA species may affect intracellular pathways in the recipient cells, contributing to the overall response to EV treatment. Finally, we are aware that positive results obtained in a preclinical animal model do not always translate into effective therapy in human patients. Thus, further efforts are needed to fully explore the activity of hiPS-EVs. This study opens new perspectives in both basic and translational sciences.

### Supplementary Information

**Additional file 1: Figure S1.** Microscopic analysis of two human induced pluripotent stem cell (hiPSC) lines (L2 and L3) cultured in different oxygen conditions: N - atmospheric oxygen concentration - normoxia; H5 - hypoxia 5% O_2_; H3 - hypoxia 3% O_2_. **Figure S2.** Optimization of isolation of hiPS-EVs. Two methods were compared: ultrafiltration combined with size-exclusion chromatography (UF+SEC) and ultracentrifugation (UC). In the UF+SEC method, filter tubes with different levels of protein cut-off based on size were used: 10, 50 and 100 kDa. **Figure S3.** The effect of EV dose and type from different oxygen concentration on human cardiac fibroblasts (hCFs). **Figure S4.** Analysis of the impact of hiPS-EV-H5 on the actin cytoskeleton, focal contacts and mechanical properties of human cardiac fibroblasts (hCFs) stimulated with TGFβ (1 ng/ml). EV-H5 from hiPSC-L1 and 2 were used. **Figure S5.** Analysis of fibrosis-related signaling pathway involving SMAD2 and SNAI2 transcription factors in human cardiac fibroblasts (hCFs) stimulated with TGFβ (1 ng/ml) and treated with EV-H5 (from hiPS-L1 and 2).

## Data Availability

All data supporting reported results are available upon reasonable request from the corresponding author.

## Abbreviations

α-SMA: Alpha smooth muscle actin
AFM: Atomic force microscopy
BSA: Bovine serum albumin
CM: Conditioned medium
CPCs: Cardiac progenitor cells
ESCs: Embryonic stem cells
EV-H3: EVs derived from hiPSCs cultured under 3% O_2_
EV-H5: EVs derived from hiPSCs cultured under 5% O_2_
EV-N: EVs derived from hiPSCs cultured under normoxia, 21% O_2_
EVs: Extracellular vesicles
FBS: Fetal bovine serum
FMT: Fibroblast-to-myofibroblast transition
GO: Gene ontology
H&E: Hematoxylin and eosin
hCFs: Human cardiac fibroblasts
hDFs: Human dermal fibroblasts
hiPSCs: Human induced pluripotent stem cells
MSCs: Mesenchymal stem/stromal cells
PBS: Phosphate buffered saline
RAAS: The renin–angiotensin–aldosterone system
RFU: Relative fluorescence units
RT: Room temperature
SEC: Size-exclusion chromatography
TEM: Transmission electron microscopy
TGFβ: Transforming growth factor beta
UC: Ultracentrifugation
UF: Ultrafiltration
