# Supplementary material for: Hypoxia enhances anti-fibrotic properties of extracellular vesicles derived from hiPSCs via the miR302b-3p/TGFβ/SMAD2 axis
Source: BMC Med. 2023 Oct 31;21:412. doi: 10.1186/s12916-023-03117-w (PMC10617123; doi:10.1186/s12916-023-03117-w)
Supplement: Supplementary file 1 — Additional file 1: Figure S1. Microscopic analysis of two human induced pluripotent stem cell (hiPSC) lines (L2 and L3) cultured in different oxygen conditions: N - atmospheric oxygen concentration - normoxia; H5 - hypoxia 5% O2; H3 - hypoxia 3% O2. Figure S2. Optimization of isolation of hiPS-EVs. Two methods were compared: ultrafiltration combined with size-exclusion chromatography (UF+SEC) and ultracentrifugation (UC). In the UF+SEC method, filter tubes with different levels of protein cut-off based on size were used: 10, 50 and 100 kDa. Figure S3. The effect of EV dose and type from different oxygen concentration on human cardiac fibroblasts (hCFs). Figure S4. Analysis of the impact of hiPS-EV-H5 on the actin cytoskeleton, focal contacts and mechanical properties of human cardiac fibroblasts (hCFs) stimulated with TGFβ (1 ng/ml). EV-H5 from hiPSC-L1 and 2 were used. Figure S5. Analysis of fibrosis-related signaling pathway involving SMAD2 and SNAI2 transcription factors in human cardiac fibroblasts (hCFs) stimulated with TGFβ (1 ng/ml) and treated with EV-H5 (from hiPS-L1 and 2). [file 12916_2023_3117_MOESM1_ESM.pdf]

Additional File 1

Hypoxia enhances anti-fibrotic properties of extracellular vesicles derived from hiPSCs *via* the miR302b-3p/TGFβ/SMAD2 axis

Milena Paw et al.

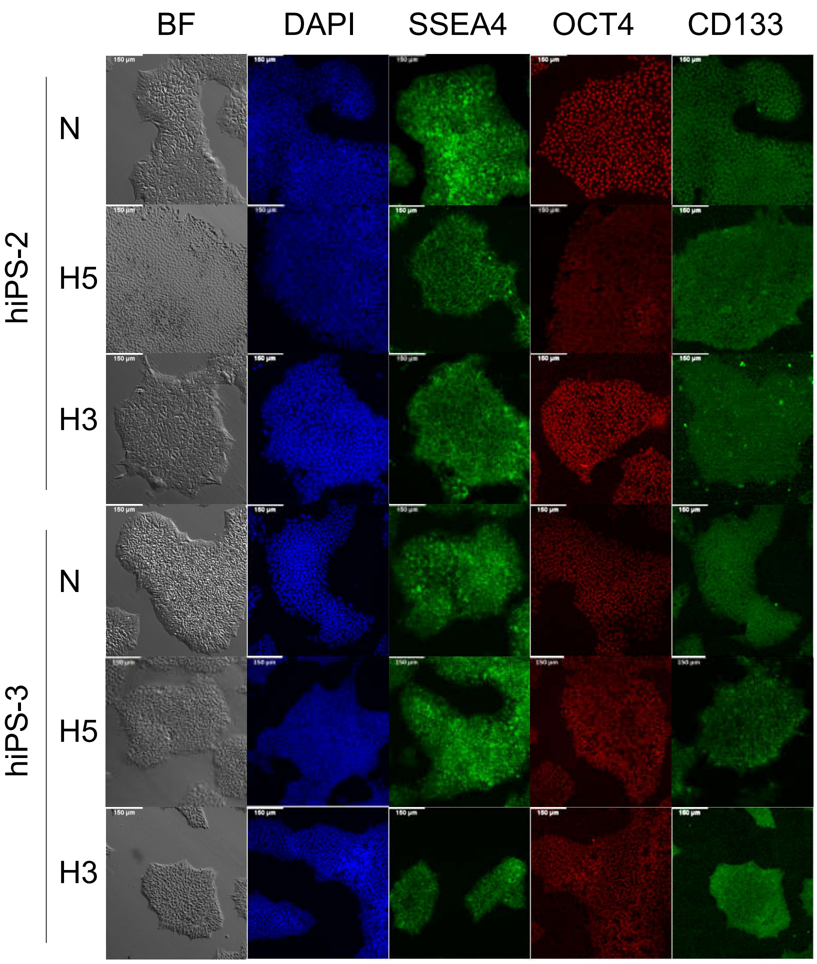

Fig. S1. Microscopic analysis of two human induced pluripotent stem cell (hiPSC) lines (L2 and L3) cultured in different oxygen conditions: N - atmospheric oxygen concentration - normoxia; H5 - hypoxia 5% O_2; H3 - hypoxia 3% O_2. hiPSC colonies shown in bright field (BF) and stained for the presence of pluripotency markers: SSEA4, OCT4 and CD133. Cell nuclei stained with DAPI. Scale bar 100 μm.

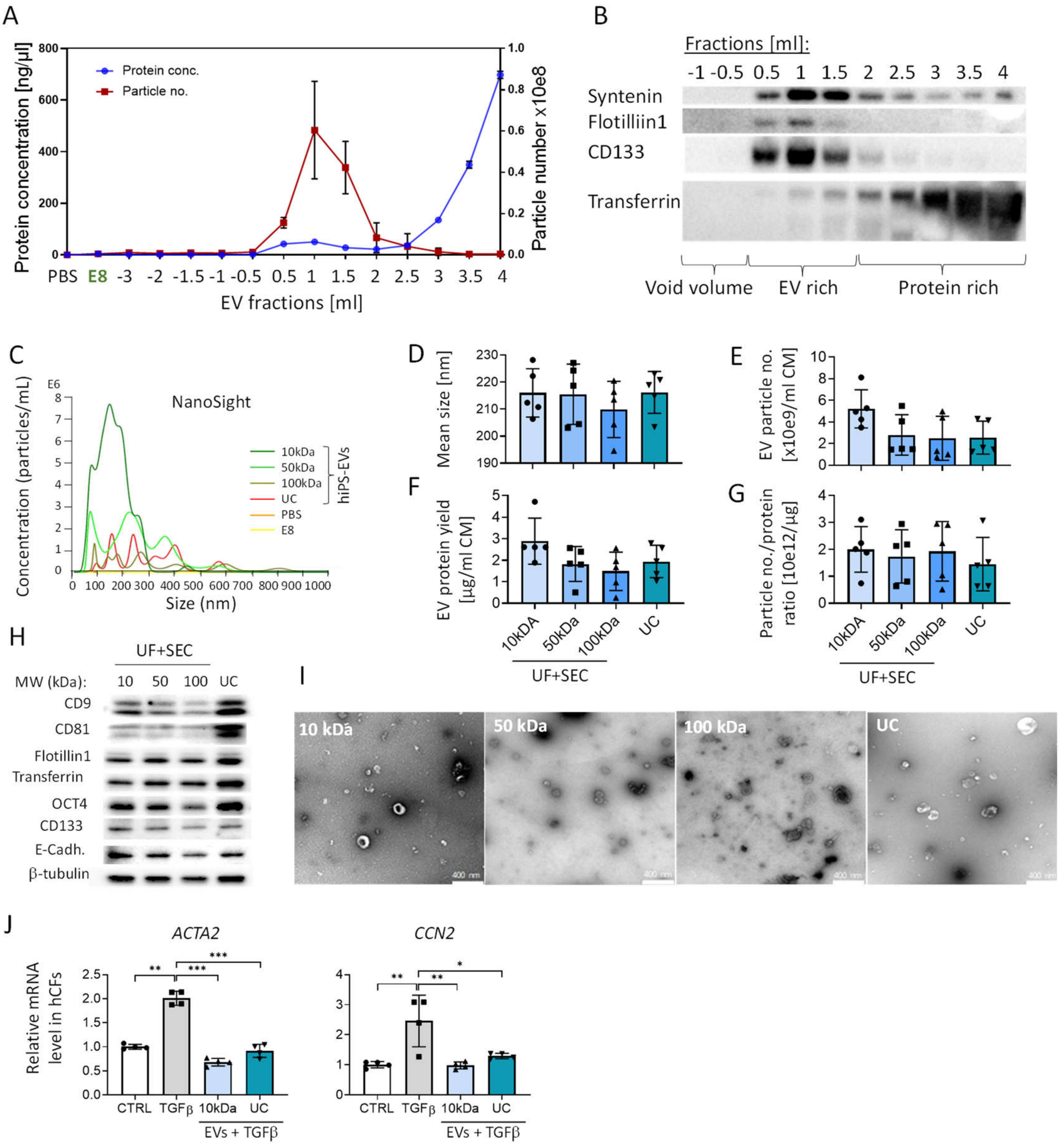
A
Protein concentration [ng/µl]
Particle number x10e8
Protein conc.
Particle no.
PBS E8 -3 -2 -1.5 -1 -0.5 0.5 1 1.5 2 2.5 3 3.5 4
EV fractions [ml]
B
Fractions [ml]:
-1 -0.5 0.5 1 1.5 2 2.5 3 3.5 4
Syntenin
Flotilliin1
CD133
Transferrin
Void volume
EV rich
Protein rich
C
Concentration (particles/mL)
NanoSight
10kDa
50kDa
100kDa
UC
PBS
E8
hiPS-EVs
Size (nm)
D
Mean size [nm]
E
EV particle no. [x10e9/ml CM]
F
EV protein yield [µg/ml CM]
10kDA
50kDa
100kDa
UC
UF+SEC
G
Particle no./protein ratio [10e12/µg]
H
UF+SEC
MW (kDa): 10 50 100 UC
CD9
CD81
Flotillin1
Transferrin
OCT4
CD133
E-Cadh.
β-tubulin
I
10 kDa
50 kDa
100 kDa
UC
J
ACTA2
CCN2
Relative mRNA level in hCFs
CTRL
TGFβ
10kDa
UC
EVs + TGFβ

Fig. S2. Optimization of isolation of hiPS-EVs. Two methods were compared: ultrafiltration combined with size-exclusion chromatography (UF+SEC) and ultracentrifugation (UC). In the UF+SEC method, filter tubes with different levels of protein cut-off based on size were used: 10, 50 and 100 kDa. **A** Analysis of particle and protein concentration in fractions obtained from hiPS-H5 conditioned medium (CM) processed with the 70qEV (Izon) column, using the NanoSight and BCA method. Samples of PBS and Essential8 medium (E8; processed with the UF-10kDa+SEC method) were used as controls (n=3). **B** Western blot analysis of proteins typical for EVs (syntenin, flotillin1), a marker of pluripotency (CD133) and a protein from cell culture medium (transferrin) in fractions obtained from the 70qEV (Izon) column. **C** Representative histogram of size and concentration of EVs obtained with different methods, measured with the NanoSight device. **D** Size analysis of EVs isolated by different methods (n=5). **E** Average particle number of EVs measured with the NanoSight device, calculated per ml of CM (n=5). **F** Analysis of the EV protein yield calculated per ml of CM (n=5). **G** Ratio of EV particle umber to protein concentration (n=5). **H** Western blot analysis of proteins typical for EVs: CD9, CD81, flotillin1, protein abundantly present in cell culture medium (transferrin), pluripotency markers (OCT4, CD133, E-cadherin), and control - β-tubulin. **I** EVs visualized by transmission electron microscopy. Representative images are shown. **J** Analysis of transcript levels of pro-fibrotic genes (*ACTA2* and *COL1A1*) in hCFs stimulated with TGFβ and treated with EVs isolated by UF-10kDa+SEC or UC method by real-time qPCR. All data are presented as the mean ± SD. Statistical significance was tested using one-way ANOVA and the Tukey post-hoc test (D, G, J: *COL1A1*), Welch ANOVA followed by a post hoc analysis with the Dunnett test (J: *ACTA2*), Kruskal-Wallis with the Dunn's test (E, F). *$p<0.05$; **$p<0.01$; ***$p<0.001$.

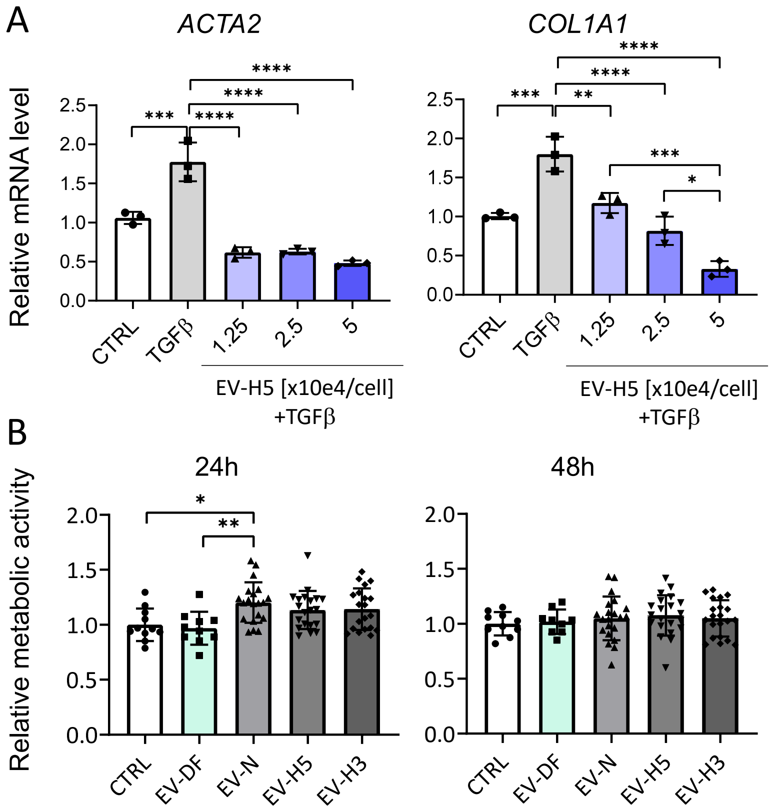

Fig. S3. The effect of EV dose and type from different oxygen concentration on human cardiac fibroblasts (hCFs). **A** Dose-dependent analysis of expression levels of pro-fibrotic genes (*ACTA2*, *COL1A1*) by real-time qPCR. EV-H5 from hiPSC-L3 were added to hCFs pre-stimulated with TGFβ (1 ng/ml) at a dose of 1.25; 2.5 and 5x10e4/cell (n=3). **B** Metabolic activity of hCFs treated with hiPS-EVs at 24 h and 48 h, relative to control (CTRL) (n=10: CTRL, EV-DF; n=20: EV-N, H5, H3). All data are presented as the mean ± SD. Statistical significance was tested using one way ANOVA with the Tukey's post-hoc test (A, B). *p<0.05; **p<0.01; ***p<0.001; ****p<0.0001.

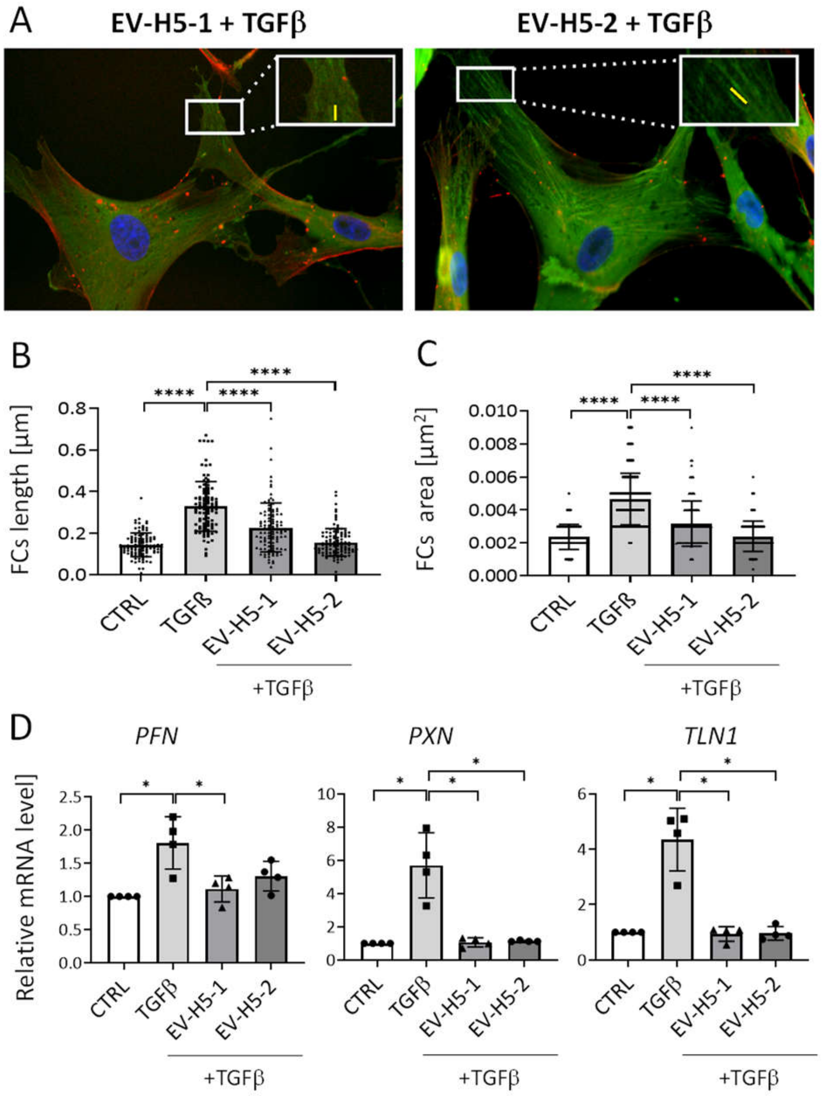

Fig. S4. Analysis of the impact of hiPS-EV-H5 on the actin cytoskeleton, focal contacts and mechanical properties of human cardiac fibroblasts (hCFs) stimulated with TGFβ (1 ng/ml). EV-H5 from hiPSC-L1 and 2 were used. **A** Representative fluorescence microscopy images of hCFs treated with EV-H5. The insets in the upper corners of the images are zoomed areas marked with white rectangles showing mature focal contacts (FCs). Quantitative data of FCs measurements are shown in graphs below: FCs length (**B**) and area (**C**) (n=105). **D** Analysis of transcript levels of genes associated with the formation of FCs (*PFN, PXN, TLN1*) by real-time qPCR (n=4). All data are presented as the mean ± SD. Data for control (CTRL) and TGFβ-treated samples shown in graphs B-D, are the same as in the main Fig. 4B-D. Statistical significance was tested using Kruskal-Wallis with the Dunn's post-hoc test (B-D). *p<0.05; **p<0.01; ***p<0.001; ****p<0.0001.

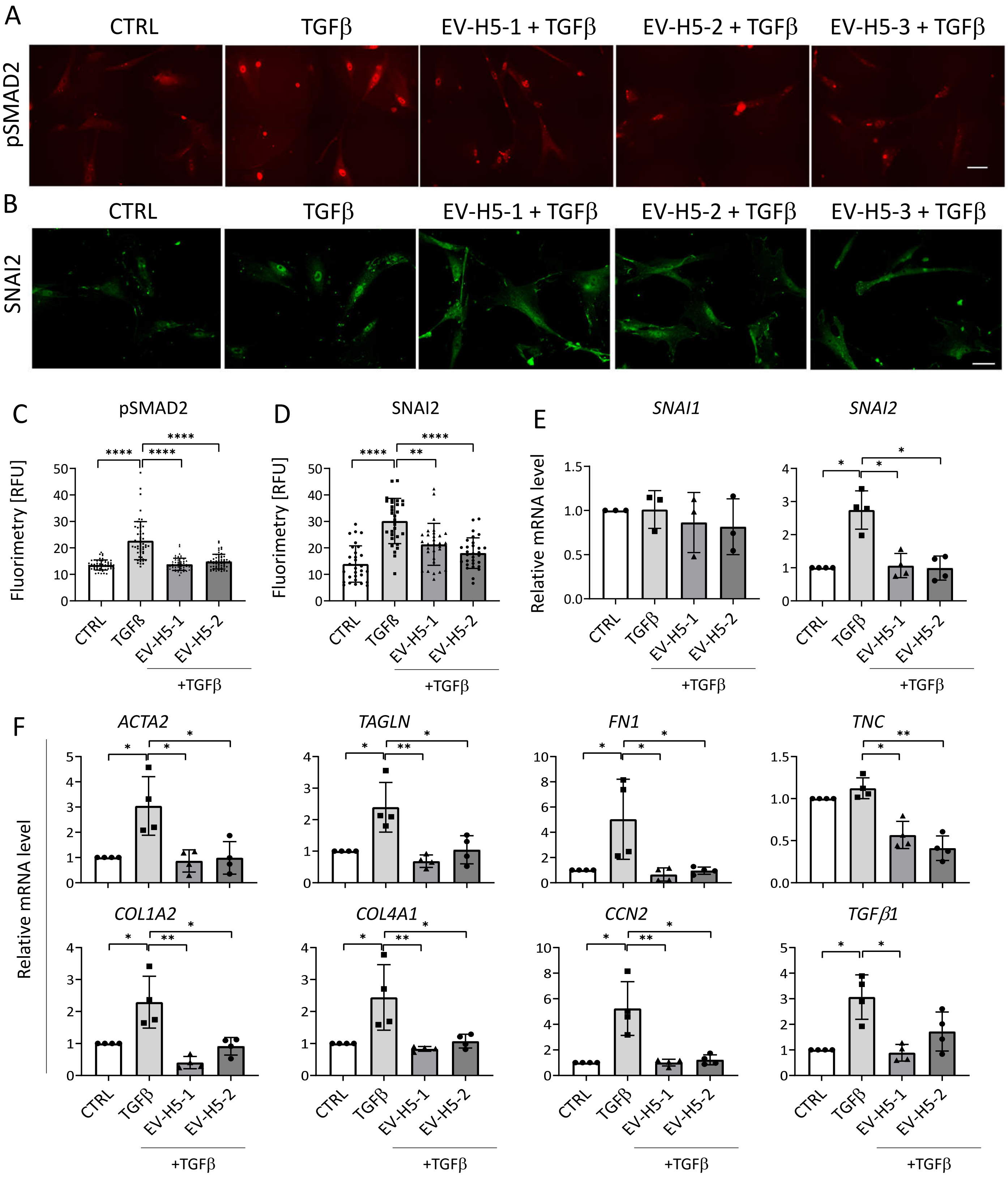
A
CTRL
TGFβ
EV-H5-1 + TGFβ
EV-H5-2 + TGFβ
EV-H5-3 + TGFβ
pSMAD2
B
SNAI2
C
pSMAD2
Fluorimetry [RFU]
D
SNAI2
E
SNAI1
SNAI2
Relative mRNA level
F
ACTA2
TAGLN
FN1
TNC
COL1A2
COL4A1
CCN2
TGFβ1
EV-H5-1
EV-H5-2
+TGFβ

Fig. S5. Analysis of fibrosis-related signaling pathway involving SMAD2 and SNAI2 transcription factors in human cardiac fibroblasts (hCFs) stimulated with TGF-β (1 ng/ml) and treated with EV-H5 (from hiPS-L1 and 2). Immunofluorescent staining of pSMAD2 (**A**) and SNAI2 (**B**) proteins in hCFs. Fluorimetric analysis of pSMAD2 (n=55) (**C**) and SNAI2 (n=30) (**D**) protein level in cell nuclei. **E** Relative quantification of *SNAI1* (n=3) and *SNAI2* (n=4) genes expression in the tested samples by real-time qPCR. **F** Analysis of mRNA levels of pro-fibrotic genes in activated hCFs (n=4). All data are presented as the mean ± SD. . Data for control (CTRL) and TGFβ-treated samples shown in graphs C-F are the same as in the main Fig. 5D-G. Statistical significance was tested using Kruskal-Wallis with the Dunn's post-hoc test (C-F). *p<0.05; **p<0.01; ****p<0.0001.
